# VirusPredictor: XGBoost-based software to predict virus-related sequences in human data

**DOI:** 10.1093/bioinformatics/btae192

**Published:** 2024-04-10

**Authors:** Guangchen Liu, Xun Chen, Yihui Luan, Dawei Li

**Affiliations:** Department of Microbiology and Molecular Genetics, University of Vermont, Burlington, Vermont 05405, United States; School of Mathematics, Shandong University, Jinan, Shandong 250100, China; School of Mathematics and Statistics, Ludong University, Yantai, Shandong 264025, China; Department of Microbiology and Molecular Genetics, University of Vermont, Burlington, Vermont 05405, United States; School of Mathematics, Shandong University, Jinan, Shandong 250100, China; Department of Microbiology and Molecular Genetics, University of Vermont, Burlington, Vermont 05405, United States; Department of Immunology and Molecular Microbiology, Texas Tech University Health Sciences Center, Lubbock, Texas 79430, United States; ICanCME Research Network, Sainte-Justine University Hospital Research Center, Montreal, Quebec H3T 1C5, Canada

## Abstract

**Motivation:**

Discovering disease causative pathogens, particularly viruses without reference genomes, poses a technical challenge as they are often unidentifiable through sequence alignment. Machine learning prediction of patient high-throughput sequences unmappable to human and pathogen genomes may reveal sequences originating from uncharacterized viruses. Currently, there is a lack of software specifically designed for accurately predicting such viral sequences in human data.

**Results:**

We developed a fast XGBoost method and software VirusPredictor leveraging an in-house viral genome database. Our two-step XGBoost models first classify each query sequence into one of three groups: infectious virus, endogenous retrovirus (ERV) or non-ERV human. The prediction accuracies increased as the sequences became longer, i.e. 0.76, 0.93, and 0.98 for 150–350 (Illumina short reads), 850–950 (Sanger sequencing data), and 2000–5000 bp sequences, respectively. Then, sequences predicted to be from infectious viruses are further classified into one of six virus taxonomic subgroups, and the accuracies increased from 0.92 to >0.98 when query sequences increased from 150–350 to >850 bp. The results suggest that Illumina short reads should be *de novo* assembled into contigs (e.g. ∼1000 bp or longer) before prediction whenever possible. We applied VirusPredictor to multiple real genomic and metagenomic datasets and obtained high accuracies. VirusPredictor, a user-friendly open-source Python software, is useful for predicting the origins of patients’ unmappable sequences. This study is the first to classify ERVs in infectious viral sequence prediction. This is also the first study combining virus sub-group predictions.

**Availability and implementation:**

www.dllab.org/software/VirusPredictor.html.

## 1 Introduction

Despite remarkable advances in high-throughput sequencing (HTS) methods, there remain many human infectious viruses that have not yet been identified or characterized (Kowarsky *et al.* 2017). These uncharacterized viruses may contribute to the etiopathology of some “unsolved” human diseases, such as myalgic encephalomyelitis/chronic fatigue syndrome (ME/CFS), where a viral origin has long been speculated but not conclusively established. Approximately 1%–15% (Chen and Li 2021) of the Illumina whole-genome sequencing short reads cannot be aligned to the human reference genome or existing viral genomes. While some of these unmapped reads may be attributed to genetic variations, repetitive regions, sequencing errors, or contaminations (Chen and Li 2021), the remainder could harbor sequences from yet-to-be-identified pathogens. Experimentally screening each of these sequences is both costly and impractical. HTS data has been generated for these “unsolved” diseases and made publicly available, e.g. whole-genome, whole-transcriptome, and metagenomic sequencing which captured all sequences present in patient biosamples, including sequences derived from any DNA and RNA viruses. Of clinical interest, patient derived HTS data can be used to computationally discover a short list of pathogen sequences that may be related to disease etiology, which can then be verified experimentally. Therefore, re-analyzing the existing patient HTS unmappable sequences will allow us to investigate possible viral associations or causalities in diseases with unclear etiologies. This study is motivated by the need to develop a computational approach capable of predicting viral sequences from unmappable sequence data.

There are two primary categories of viral sequence detection approaches: alignment-based and alignment-free. Alignment-based approaches, e.g. BLASTN, rely on sequence homology for membership inference, making them successful in many applications (Roux *et al.* 2011) but less sensitive to divergent viral sequences with rearrangements, e.g. genetic recombination, shuffling, and horizontal gene transfer, which are common in various viral genomes (Borozan *et al.* 2015). Uncharacterized and emerging viruses typically have no reference genomes available for alignment. Another limitation of alignment-based approaches is the intense computational capacity required to analyze large datasets (Vinga and Almeida 2003, Borozan *et al.* 2015), which are becoming increasingly common. In contrast, alignment-free approaches offer different advantages, e.g. they involve comparisons of sequence features such as word patterns and frequencies of sequences (Song *et al.* 2014), and thus are not affected by genome rearrangements (Leimeister *et al.* 2014); and their runtime is usually linear in proportion to sequence lengths (Vinga and Almeida 2003, Leimeister *et al.* 2014), allowing for more efficient big data analyses.

Machine learning techniques, particularly tree-based ensemble classifiers consisting of multiple decision trees, have greatly improved data processing capabilities in recent years. The eXtreme Gradient Boosting (XGBoost) (Chen and Guestrin 2016) is a state-of-the-art ensemble machine learning algorithm known for its scalability and effectiveness in classification and regression problems. “Boosting” is an effective and widely used model training algorithm that can continuously improve and integrate classifiers to create a more accurate classifier. XGBoost uses additive models and forward distribution algorithms to form an ensemble tree with multiple classification and regression tree-based learners, and then scores multiple trees based on the “higher score wins” principle to achieve target value prediction. XGBoost has the advantages of fast parallel processing, high performance, scalability to big data, and the ability to handle sparse training data. XGBoost has been applied to various biomedical fields, such as gene-gene interactions (Guo *et al.* 2021), diabetes detection (Paleczek *et al.* 2021), and prediction of COVID-19 vaccine administration priorities (Romeo and Frontoni 2022), yet, XGBoost has not been explored to detect uncharacterized or emerging viruses from human sequence data.

One of the unique challenges in predicting viral sequences in HTS data is the presence of >600 000 endogenous retrovirus (ERV) sequences, comprising 8% of the human genome. ERVs resulted from the fixation of ancient retroviral infections and integrations into the human genome. Thus, although functionally distinct, ERVs share high sequence similarities with exogenous infectious viruses, particularly retro-transcribing viruses and other single-stranded RNA viruses. However, ERVs are not considered in infectious viral sequence prediction, which may be problematic. In this study, we introduce a novel XGBoost framework, leveraging our in-house viral genome database, to predict viral-related sequences, including ERVs, in patient’s unmappable sequences.

## 2 Materials and methods

### 2.1 Datasets and preprocessing

To develop the three-class virus prediction XGBoost model (i.e. to classify among infectious virus, human ERV, and non-ERV human sequences), we first curated the infectious viral sequences based on two databases: our in-house viral reference genome database (Chen *et al.* 2019) containing 377 009 unique sequences with a minimum length of 100 base pair (bp) and the NCBI Viral Genomes database containing 13 273 complete genome sequences. Combining the two databases led to a total of 390 282 viral sequences utilized in this study. We then extracted ERV sequences from the human genome reference GRCh38.p13 using bedtools *getfasta* function and kept 614 316 ERV sequences with a minimum length of 100 bp. Our non-ERV human sequences were compiled from two sources: the human GRCh38.p13 reference genome after we masked all ERV sequences using bedtools *maskfasta* function (mitochondrial sequences included), and the NCBI Consensus Coding Sequence database containing 33 408 sequences with a minimum length of 100 bp. To develop the six-class subgroup prediction XGBoost model (i.e. to classify among six virus taxonomy subgroups), we utilized the 377 009 partial and whole genomes from our in-house viral genome database, each having been annotated according to the Baltimore classification, i.e. double-strand DNA (dsDNA), single-strand DNA (ssDNA), retro-transcribing (Retro), negative-sense single strand RNA [ssRNA (−)], positive-sense single strand RNA [ssRNA (+)], and double-strand RNA (dsRNA). All sequence data are stored in FASTA format in our pipeline.

To satisfy our model requirements, we performed three data preprocessing procedures. First, we only retained sequences with lengths ≥100 bp, which was necessary for feature extraction, also in part because most Illumina HTS read lengths are ≥100 bp. Second, we filtered out sequences with ambiguous nucleotides “N”. Third, to train our models for classifying various sequence lengths, particularly assembled contigs, we created additional training datasets by randomly cutting our training datasets (i.e. 95% of the total datasets) by 10 different length gradients (i.e. 100, 150, 200, 300, 500, 700, 1000, 1500, 2000, and 3000 bp), which cover the sequence lengths of, for instance, Illumina short read sequencing (150–350 bp), Sanger sequencing (850–950 bp), and assembled contigs (we examined sequences of 2000–5000 bp, but not longer, because our results have already shown very high accuracies when sequences were >1000 bp). To balance the number of sequences among all the examined groups, we controlled the amounts of the sequences from the length gradients and then merged them with the original sequences. After the preprocessing, for our three-class virus prediction model, we obtained 1 489 300 viral, 1 515 342 ERV, and 1 578 686 non-ERV sequences, with 8838 sequences for testing and the rest for model training (Supplementary Table S1); and for our six-class subgroup prediction model, we obtained a total of 1 153 837 viral sequences, with 8387 sequences for testing and the rest for training (Supplementary Table S2).

### 2.2 XGBoost classification models

**Figure 1.**
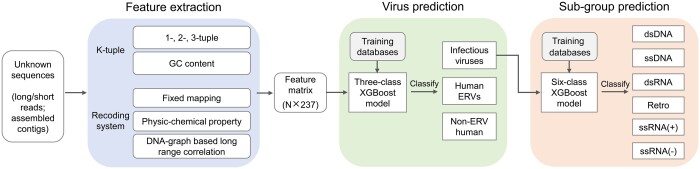
Flowchart of VirusPredictor sequence prediction pipeline. *N* is the number of input sequences.

Using our preprocessed training datasets, we developed XGBoost-based classification models, which showed the best performance compared to other machine learning algorithms (described below). Our trained XGBoost models have three distinct functions; first, the training sequences are transformed into numerical vectors, and a total of 237 informative features of the sequences are extracted via the K-tuple relative abundance and recoding system; second, our virus prediction model classifies each testing sequence into one of the three groups (infectious virus, ERV or non-ERV human); third, our subgroup prediction model then further classifies each viral sequence into one of six taxonomy subgroups. Figure 1 depicts the flowchart of our method and software package (VirusPredictor). The following paragraphs illustrate the development process of the models and software.

#### 2.2.1 Sequence feature extraction

To extract informative numerical features from the training sequences, we first transformed each DNA sequence into a numerical sequence using the alignment-free K-tuple (Ning *et al.* 2001) and recoding system (Supplementary Methods S1.1.1). We extracted 237 informative features using relative abundance (Supplementary Methods S1.1.2 formulas S2 and S3) and DNA spectral analyses (Kwan *et al.* 2012). Supplementary Fig. S1 illustrates our sequence transform and feature extraction strategy. Supplementary Fig. S2 depicts the hierarchical structure of methods for our feature extraction procedures. Supplementary Table S3 summarizes the specific methods used in the recoding system.

#### 2.2.2 Selections of machine learning algorithms and features

To determine the best machine learning algorithm, we developed multiple classification models using four different algorithms, including random forest, K nearest neighbor, support vector machine, and XGBoost. We then compared the performance among the four algorithms using the same training and testing files, and comparable procedures (e.g. tuning hyperparameters for the optimal performance). To determine the minimum number of informative features, we implemented feature engineering procedures followed by comparing the performance of various numbers of features, i.e. we computed an importance score for each feature using random forest (Yu *et al.* 2021), and then selected the top 20, 50, 100 important features and compared their prediction performance with when all features were used. The procedures are described in Supplementary Methods S1.1.3.

#### 2.2.3 Summary of our two-step XGBoost models

Our XGBoost models contain two steps: three-class virus prediction followed by six-class subgroup prediction. The details of our XGBoost models are described in Supplementary Methods S1.2.1 and S1.2.2. In our virus prediction model, our testing data included three length gradients, including 2938 sequences of 150–350 bp (Illumina short reads), 2937 sequences of 850–950 bp (Sanger sequencing), and 2963 sequences of 2000–5000 bp (Supplementary Table S1). In our subgroup prediction model, the testing data also contained three length gradients, including 2792 sequences of 150–350 bp, 2711 sequences of 850–950 bp, and 2884 sequences of 2000–5000 bp (Supplementary Table S2). All necessary data preprocessing procedures, e.g. normalization of training and testing data, have been considered in our analyses (see Supplementary Methods S1.2.3 for details).

#### 2.2.4 Model performance assessment

Our two-step prediction models from each of the four machine learning algorithms can be considered as a collection of multiple binary classifications. To assess the performance of each trained model, we adopted the macro average of binary metrics, including macro average accuracy, recall, precision, and F1-score (Supplementary Methods S1.3), which provides equal weight to these binary classes (Sokolova and Lapalme 2009).

#### 2.2.5 VirusPredictor software development

To implement our two-step XGBoost models, we developed a new software package named VirusPredictor. The software first transforms query sequences into a feature matrix where 23 different methods are utilized to generate a total of 237 features for each input sequence. Then, our trained virus prediction model classifies sequences among infectious virus, ERV, and non-ERV human groups. Our trained subgroup prediction model further classifies each predicted viral sequence into subgroups. Users can modify the algorithms and/or parameters for advanced analyses. VirusPredictor is written in Python and is open-source and freely available (www.dllab.org/software/VirusPredictor.html).

## 3 Results

### 3.1 Machine learning algorithms and features

**Figure 2.**
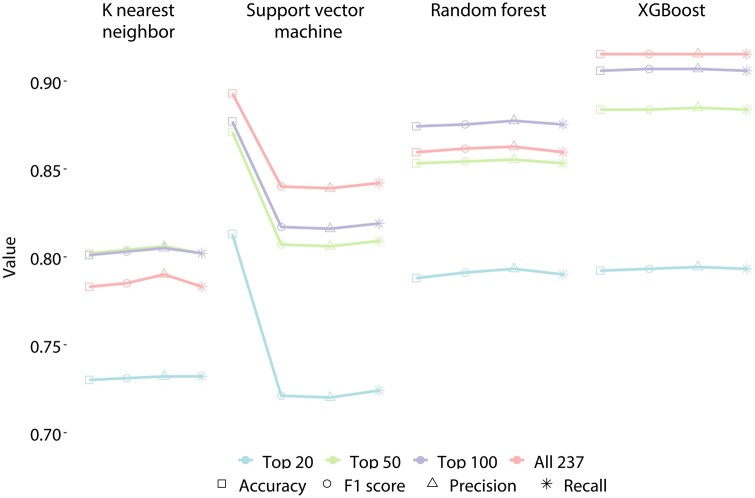
Performance comparisons of four machine learning algorithms and different features. Macro average accuracy, F1-score, precision, and recall are shown.

A total of 237 numerical features were extracted for each training sequence regardless of its original length. We sorted the 237 features according to their relative importance (Supplementary Table S4). We compared the performance to predict infectious virus, ERV, and non-ERV human sequences among the four algorithms, including random forest, K nearest neighbor, support vector machine, and XGBoost, as well as different numbers of features using 302 169 sequences, a subset randomly selected from our whole sequence data, with 15 108 sequences used for testing and the remaining for model training. Our results showed that the models achieved higher accuracy and F1-scores when more features were included; and XGBoost outperformed almost all the other machine learning algorithms with the highest accuracies using identical data and procedures. Among all the combinations, XGBoost with all 237 features resulted in the best performance, i.e. 0.904 macro average accuracy and 0.904 F1-score (Fig. 2 and Supplementary Table S5). Therefore, we then utilized our whole sequence database to train the two-step XGBoost models using the entire 237 features.

### 3.2 Three-class XGBoost model to identify viral-related sequences

**Figure 3.**
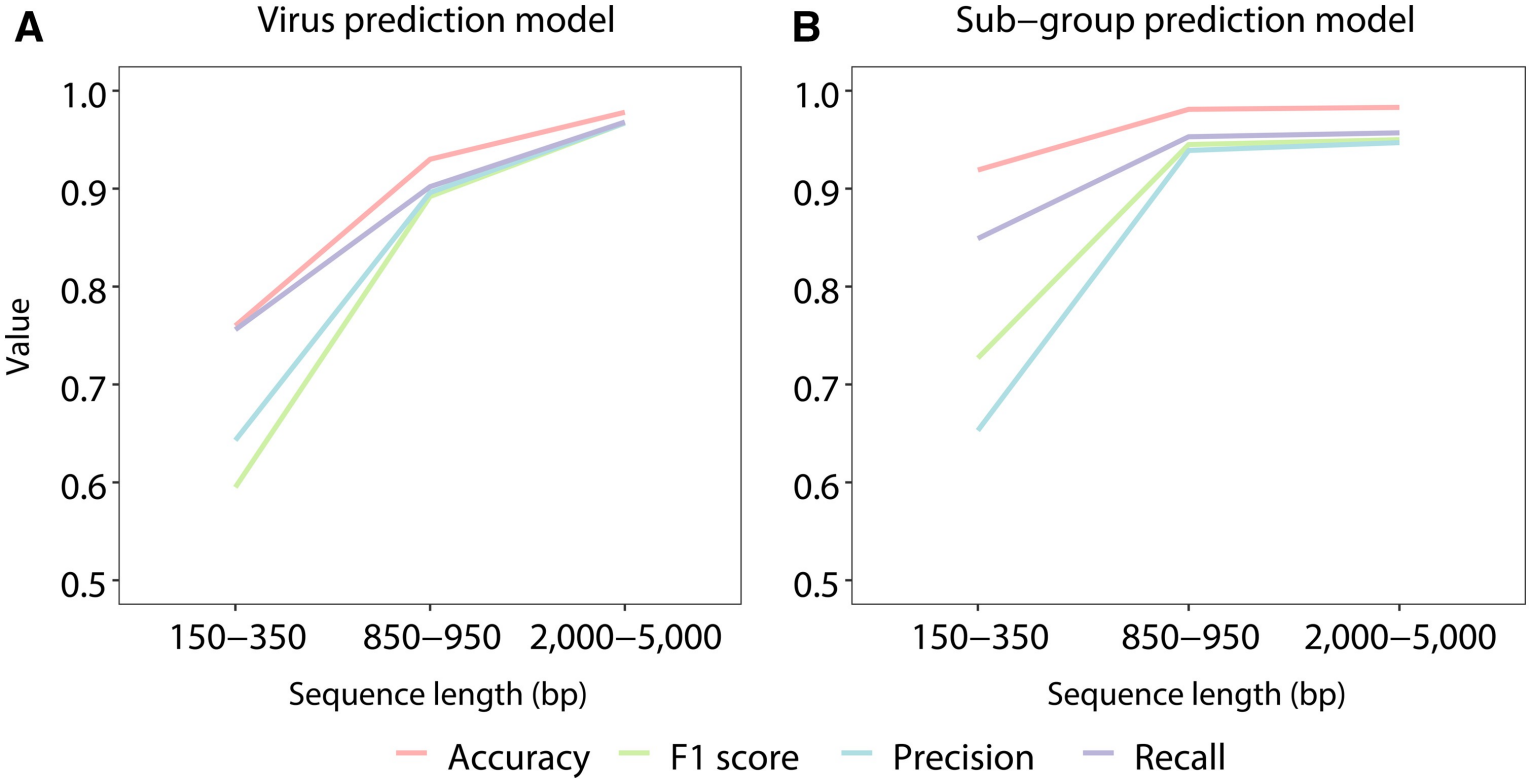
Performance of (A) our three-class virus prediction and (B) six-class subgroup prediction XGBoost models. The sequence lengths 150–350 and 850–950 bp represent the typical ranges of Illumina short reads and Sanger sequencing data, respectively. Macro average accuracy, F1-score, precision, and recall are shown.

**Figure 4.**
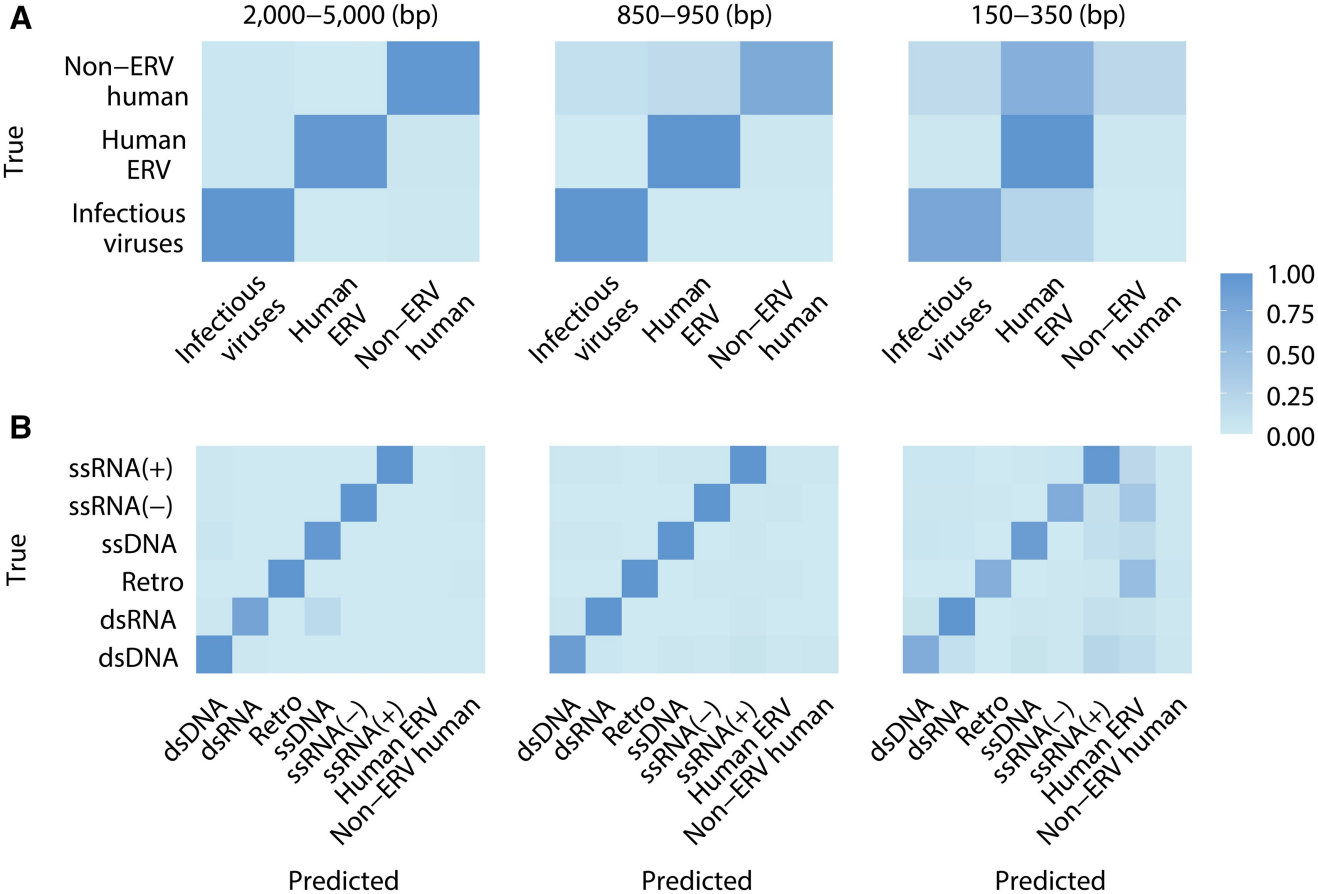
Heatmaps of pair-wise confusion matrix of (A) our virus prediction and (B) subgroup prediction XGBoost models. The value in each cell is the ratio of predicted number divided by the total number of testing sequences of each row (Supplementary Tables S7 and S9). The cells on the diagonal represent correct predictions.

Our three-class virus prediction XGBoost model performance metrics increased as the lengths of the testing sequences increased. The macro average accuracies were 0.76 and 0.93 when the testing sequences were 150–350 bp and 850–950 bp, respectively. When the testing sequences were 2000–5000 bp (e.g. assembled long contigs), the prediction accuracies further increased with average accuracy 0.978, recall 0.968, precision 0.967, and F1-score 0.967 (Fig. 3A and Supplementary Table S6). The pair-wise confusion matrix and model performance metrics of the virus prediction XGBoost model are shown in Fig. 4A and Supplementary Table S7.

### 3.3 Six-class XGBoost model to predict virus taxonomic subgroups

To classify each identified viral sequence into one of the six virus taxonomic subgroups, we further trained a six-class XGBoost classifier using our in-house viral database. After hyperparameter tuning, the trained model showed high accuracies in classifying among the six subgroups, i.e. dsDNA, ssDNA, Retro, ssRNA(−), ssRNA(+), and dsRNA. When the testing sequences were 150–350 bp, the macro average accuracy and F1-score were 0.919 and 0.727, respectively. When the testing sequences were 850–950 bp and 2000–5000 bp, the accuracy and F1-score reached > 0.98. Considering that each of the six classes only has ∼0.16 probability of being correctly classified at random, our model has high performance in predicting viral subgroups (Fig. 3B and Supplementary Table S8). The pair-wise confusion matrix of the subgroup prediction model is shown in Fig. 4B and Supplementary Table S9. The optimal hyperparameter combinations used in our models are shown in Supplementary Table S10.

### 3.4 Applications of VirusPredictor software

**Figure 5.**
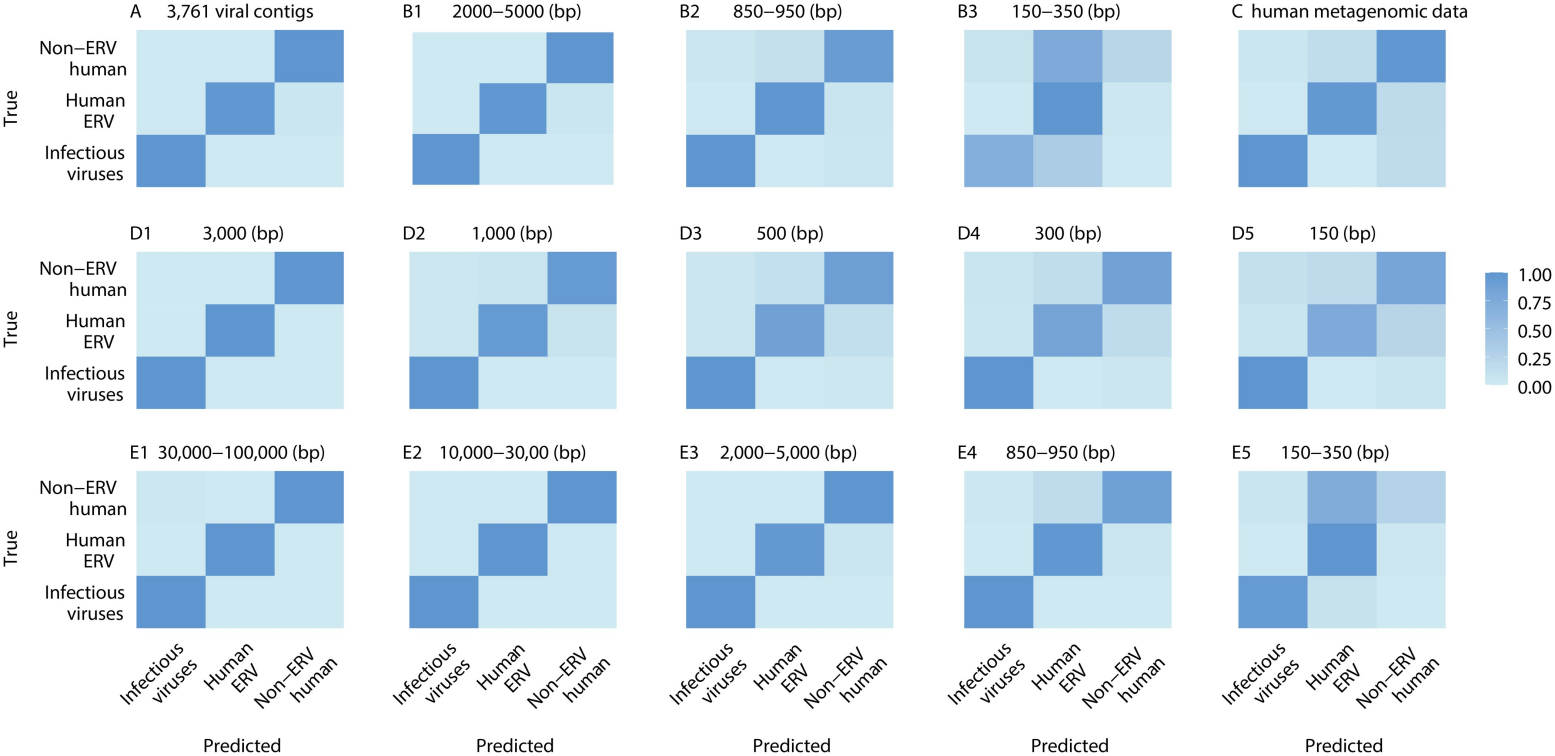
Heatmaps of pair-wise confusion matrix of our virus prediction XGBoost model to predict (A) 3761 viral contigs, (B1–B3) SARS-CoV-2 sequences, (C) Illumina 300 bp short reads from human metagenomic data, (D1–D5) phage sequences, and (E1–E5) DNA viruses from human gut microbiome. The value in each cell is ratio of predicted number divided by total number of testing sequences of each row (Supplementary Tables S11, S13–S16). The cells on the diagonal represent correct predictions.

We developed a novel Python software, VirusPredictor, to implement our two-step XGBoost models. We then applied VirusPredictor to five publicly available real viral sequence datasets and obtained high prediction accuracies. For example, a study (Kowarsky *et al.* 2017) identified 3761 contigs, validated by polymerase chain reactions, of uncharacterized microbes colonized in human circulating cell-free DNA. We used VirusPredictor to predict these contigs (average length 2595 bp with minimum 1000 bp and maximum 105 439 bp). Our results showed that 3747 of the 3761 contigs were predicted as viral sequences, suggesting 0.996 accuracy. We then added 4000 ERV and 4000 non-ERV human sequences randomly selected from the whole human genome (i.e. the entire ERV and non-ERV human sequences, respectively), and repeated the prediction. We obtained 0.967–0.996 sensitivity, 0.981–0.997 specificity, 0.965–0.993 precision, 0.003–0.019 false positive rates, and 0.986–0.997 accuracies to predict among virus, ERV, and non-ERV human sequences (Supplementary Table S11 and Fig. 5A).

In our second application, we used VirusPredictor to predict the severe acute respiratory syndrome coronavirus 2 (SARS-CoV-2) genome (NC_045512.2; 29 903 bp). When the whole SARS-CoV-2 genome sequence was used as input, VirusPredictor predicted it to be of viral origin, and then accurately classified it as a ssRNA(+) virus. To challenge VirusPredictor, we randomly extracted 300 continuous fragments from the SARS-CoV-2 genome with three length gradients (150–350, 850–950, and 2000–5000 bp), and then used VirusPredictor to predict each of the 300 fragments. The results showed that when the SARS-CoV-2 fragments were 150–350 bp, 69% of the fragments were predicted as being a virus, while the other 31% were classified as ERVs, which admittedly share high sequence similarities with various infectious viral genomes, including the SARS-CoV-2 genome; and 80% of the viral sequences were further predicted as ssRNA(+) (the random rate is 16.7%). When the fragments were 850–950 bp, 97% were predicted as viral sequences; and 88% of the viral sequences were further predicted as ssRNA(+). When the fragments were 2000–5000 bp, 100% were predicted as virus; and 96% were further predicted as ssRNA(+) (Supplementary Table S12). We then added 300 ERV and 300 non-ERV human sequences randomly selected from the whole genome and repeated the prediction for each length group. We obtained 0.9–1 sensitivity, 0.935–1 specificity, 0.881–1 precision, 0–0.065 false positive rates, and 0.943–1 accuracies to predict among virus, ERV, and non-ERV human when the sequence lengths were >850 bp (Supplementary Table S13 and Fig. 5B).

In our third application, we applied VirusPredictor to directly predict the 5551 Illumina short reads (300 bp) of potential viral sequences from 19 metagenomic datasets (Tampuu *et al.* 2019) after we randomly checked by BLAST that the vast majority of these reads map to viruses. Our results showed that 83.3% of these short reads were predicted as virus. We then added 5600 ERV and 5600 non-ERV human sequences (300 bp) randomly selected from the whole genome and repeated the prediction. We obtained 0.806–0.833 sensitivity, 0.819–0.957 specificity, 0.694–0.906 precision, 0.043–0.18 false positive rates, and 0.818–0.916 accuracies to predict among virus, ERV, and non-ERV human (Supplementary Table S14 and Fig. 5C).

In our fourth application, we applied VirusPredictor to predict the viruses (mostly phages) used in an existing study (Ren *et al.* 2020). After downloading the complete genomes (mean = 70 865 bp with min = 4184 and max = 348 113) from the NCBI viral genome database and removing sequences containing missing nucleotides, we used VirusPredictor to predict the cleaned 674 genomes. All of them were classified to be viruses. We then randomly fragmented these 674 genomes into 5000 sequences with five lengths (i.e. 150, 300, 500, 1000, and 3000 bp, the same lengths as in the Ren *et al.* paper), and further added the same numbers of our ERV and non-ERV human sequences randomly selected from the whole genome. Our results showed 0.815–1 sensitivity, 0.897–1 specificity, 0.801–0.999 precision, 0.001–0.103 false positive rates, and 0.874–1 accuracies to predict among virus, ERV, and non-ERV human when the sequence lengths were >300 bp (Supplementary Table S15 and Fig. 5D).

A study (Nayfach *et al.* 2021) released the genome sequences of 189 680 under-represented DNA viruses (98% were dsDNA viruses) from the human gut microbiome. We randomly selected from these viral genomes and fragmented them into 20 000 sequences with various lengths (i.e. 150–350, 850–950, 2000–5000, 10 000–30 000, and 30 000–100 000 bp), and applied VirusPredictor. Our results showed that >99% were predicted as virus, and 81%–98.6% were further predicted into subgroups correctly when the sequence lengths were >850 bp. We then added the same numbers of randomly selected ERV and non-ERV human sequences and repeated the prediction for each length group. We obtained 0.853–1 sensitivity, 0.923–1 specificity, 0.863–1 precision, 0–0.077 false positive rates, and 0.939–1 accuracies to predict among virus, ERV, and non-ERV human when the sequence lengths were >850 bp (Supplementary Table S16 and Fig. 5E). Our study is the first of its kind classifying ERVs in viral sequence prediction. Our study is also the first to combine virus subgroup predictions.

## 4 Discussion

Many human diseases, including ME/CFS and some virus-associated cancers (Cao and Li 2018), have long been suspected to originate from viral infections, yet the identification of causative pathogens has remained difficult. Discovering viral sequences in these patients’ HTS data has been challenging, particularly for uncharacterized viruses without reference genomes. However, this is a worthwhile endeavor because unmapped sequences from patient HTS data may contain DNA/RNA derived from disease causative pathogens. In this study, we developed a fast and accurate XGBoost-based sequence screening method with user-friendly software to predict infectious viruses from human sequence data derived from long/short read genomic, transcriptomic, and metagenomic sequencing or Sanger sequencing. VirusPredictor is useful specifically for predicting the origins of sequences that cannot be aligned to existing reference genomes of species of interest. VirusPredictor can discover sequences derived from uncharacterized infectious viruses and endogenous retroviruses (for which no actual reference genomes exist), and other viral sequences that are not identifiable by alignment. We demonstrated that our XGBoost models outperformed the other machine learning algorithms (Fig. 2). Adding to user convenience, VirusPredictor accepts assembled contigs and long/short read sequences in either FASTQ or FASTA format. Since the prediction accuracies increase as the testing sequences become longer (Fig. 3), we suggest assembling short reads into contigs, e.g. ∼1000 bp (at least ∼800 bp) or longer sequences, whenever possible before predictions.

Our method has several advantages. We manually built the largest viral reference genome database and utilized it as training data in this study. We implemented a wide range of feature engineering for DNA sequences by leveraging biophysically meaningful methods, e.g. k-tuple, GC content, relative ambiguity, and genomic signal processing (Supplementary Methods S1.1, Supplementary Figs S1 and S2, and Supplementary Table S3). Thus, compared to black boxes in deep learning models, an advantage of our models is their interpretability, i.e. while obtaining high efficiency, our models can identify the key features for classifying groups (Supplementary Table S4), providing better understanding of the connections from features to predictions. Our XGBoost models are not affected by multicollinearity. Since XGBoost is based on decision trees, if two or more features are correlated, our method calculates information gain for all the features to select the optimal segmentation feature when deciding upon a splitting tree. Thus, our models are more robust than traditional multi-linear regression when handling multicollinearity. XGBoost can also prevent overfitting while improving accuracy and computational speed through multiple mechanisms (e.g. adding regular terms to objective functions, using one- and second-order Taylor expansions, adopting novel sparsity-aware algorithm, and supporting parallelism algorithm) (Chen and Guestrin 2016). Our method can be easily broadened to classify other species (e.g. bacteria, fungi, and plants), and to investigate the global diversity of species.

However, we have also recognized the limitations of this study. First, almost all machine learning algorithms cannot handle missing data with satisfactory accuracy. In this study, our software automatically checks data quality, discards sequences with missing nucleotides, and generates missing nucleotide warnings to users. Users can choose to manually remove all missing nucleotides or cut sequences into fragments at missing nucleotide junctions and use the cleaned fragments for predictions. In the latter option, as each fragment will obtain its own classification label, the final prediction can be determined by the majority of votes. Second, while it is known that the use of larger training data will lead to better predictions, the limitations of currently available CPU/GPU power led to only a fraction (∼8.9%) of the (non-ERV) human genome being used in our model training process. We anticipate these limitations can be addressed with ongoing advancements in computational resources and collaborative efforts (we made all our source code publicly available). Our software’s prediction accuracies can further increase when more powerful computers become available, allowing for using the complete human genome or a larger portion of it for training. Third, in our virus prediction model, when the testing sequences are 150–350 bp, certain non-ERV human sequences are predicted as ERVs. This reflects the fact that many *individual* ERVs have not yet been identified or annotated in the human genome, i.e. some sequences currently known as “non-ERV” are likely ERVs. In a separate study, our group is identifying individual transposable element sequences (unpublished). Our future plans involve incorporating new transposable element sequences into our models and additional algorithms (Ren *et al.* 2017, Tampuu *et al.* 2019, Ren *et al.* 2020, Alshayeji *et al.* 2023, Elbasir *et al.* 2023, Rajkumar *et al.* 2022) to enhance the predictive power of VirusPredictor. Despite the lack of annotated full ERV sequences, when the testing sequences are >850 bp, the prediction accuracies of our software increased to >90% for all scenarios.

In summary, we developed and demonstrated a novel XGBoost-based method with user-friendly software, VirusPredictor. It is capable of efficiently screening patients’ unmappable sequence data, preferably assembled contigs or sequences longer than 1000 bp, to identify potential viral-related sequences that can then be verified experimentally. This tool is especially valuable for studying suspected viral associations or causalities in diseases with unclear etiologies. By including ERVs, this research marks a pioneering step in infectious viral sequence prediction, opening avenues for further exploration and discovery.

## Supplementary Material

btae192_Supplementary_Data

## Data Availability

URLs for data presented herein are as follows: NCBI Human Genome Resources: www.ncbi.nlm.nih.gov/projects/genome/guide/human Human genome reference GRCh38.p13: www.ncbi.nlm.nih.gov/assembly/GCF_000001405.39 Transposable element annotation hg38_rmsk_TE.gtf.gz: http://hammelllab.labsites.cshl.edu/software NCBI Consensus Coding Sequence database: https://ftp.ncbi.nlm.nih.gov/pub/CCDS NCBI Viral Genomes: www.ncbi.nlm.nih.gov/genome/viruses NCBI Viral Genomes database: https://ftp.ncbi.nlm.nih.gov/refseq/release/viral International Committee on Taxonomy of Viruses: https://talk.ictvonline.org/ictvreports/ictv_online_report Baltimore classification: http://viralzone.expasy.org/254 bedtools/getfasta: https://bedtools.readthedocs.io/en/latest/content/tools/getfasta.html VirusPredictor Python package: www.dllab.org/software/VirusPredictor.html VirusPredictor transposable element-masked human genome reference GRCh38.p13: www.dllab.org/software/VirusPredictor/hg38_rmsk_ERV.fa VirusPredictor endogenous retrovirus training data (minimum length = 100 bp): www.dllab.org/software/VirusPredictor/hg38_ERV_100bp.fa

## References

[btae192-B1] Alshayeji MH , Sindhu SC, Abed S. Viral genome prediction from raw human DNA sequence samples by combining natural language processing and machine learning techniques. Expert Syst Appl 2023;218:119641.

[btae192-B2] Borozan I , Watt S, Ferretti V. Integrating alignment-based and alignment-free sequence similarity measures for biological sequence classification. Bioinformatics 2015;31:1396–404. 25573913 10.1093/bioinformatics/btv006 PMC4410667

[btae192-B3] Cao J , Li D. Searching for human oncoviruses: histories, challenges, and opportunities. J Cell Biochem 2018;119:4897–906. 29377246 10.1002/jcb.26717

[btae192-B4] Chen T , Guestrin C. XGBoost: a scalable tree boosting system. In: *Proceedings of the 22nd ACM SIGKDD International Conference on Knowledge Discovery and Data Mining*. San Francisco, CA, USA: Association for Computing Machinery. 2016. 785–94.

[btae192-B5] Chen X , Kost J, Sulovari A et al A virome-wide clonal integration analysis platform for discovering cancer viral etiology. Genome Res 2019;29:819–30. 30872350 10.1101/gr.242529.118 PMC6499315

[btae192-B6] Chen X , Li D. Sequencing facility and DNA source associated patterns of virus-mappable reads in whole-genome sequencing data. Genomics 2021;113:1189–98. 33301893 10.1016/j.ygeno.2020.12.004 PMC7856238

[btae192-B7] Elbasir A , Ye Y, Schäffer DE et al A deep learning approach reveals unexplored landscape of viral expression in cancer. Nat Commun 2023;14:785. 36774364 10.1038/s41467-023-36336-z PMC9922274

[btae192-B8] Guo Y , Wu C, Yuan Z et al Gene-based testing of interactions using XGBoost in genome-wide association studies. Front Cell Dev Biol 2021;9:801113. 34977040 10.3389/fcell.2021.801113 PMC8716787

[btae192-B9] Kowarsky M , Camunas-Soler J, Kertesz M et al Numerous uncharacterized and highly divergent microbes which colonize humans are revealed by circulating cell-free DNA. Proc Natl Acad Sci USA 2017;114:9623–8. 28830999 10.1073/pnas.1707009114 PMC5594678

[btae192-B10] Kwan HK , Kwan BY, Kwan JY. Novel methodologies for spectral classification of exon and intron sequences. EURASIP J Adv Signal Process 2012;2012:1–14.

[btae192-B11] Leimeister C-A , Boden M, Horwege S et al Fast alignment-free sequence comparison using spaced-word frequencies. Bioinformatics 2014;30:1991–9. 24700317 10.1093/bioinformatics/btu177 PMC4080745

[btae192-B12] Nayfach S , Páez-Espino D, Call L et al Metagenomic compendium of 189,680 DNA viruses from the human gut microbiome. Nat Microbiol 2021;6:960–70. 34168315 10.1038/s41564-021-00928-6 PMC8241571

[btae192-B13] Ning Z , Cox AJ, Mullikin JC. SSAHA: a fast search method for large DNA databases. Genome Res 2001;11:1725–9. 11591649 10.1101/gr.194201 PMC311141

[btae192-B14] Paleczek A , Grochala D, Rydosz A. Artificial breath classification using XGBoost algorithm for diabetes detection. Sensors (Basel) 2021;21:4187. 34207196 10.3390/s21124187 PMC8234852

[btae192-B15] Rajkumar U et al DeepViFi: detecting oncoviral infections in cancer genomes using transformers. In: *Proceedings of the 13th ACM International Conference on Bioinformatics, Computational Biology and Health Informatics*. Northbrook, IL: Association for Computing Machinery. 2022. Article 2.

[btae192-B16] Ren J , Ahlgren NA, Lu YY et al VirFinder: a novel k-mer based tool for identifying viral sequences from assembled metagenomic data. Microbiome 2017;5:69. 28683828 10.1186/s40168-017-0283-5 PMC5501583

[btae192-B17] Ren J , Song K, Deng C et al Identifying viruses from metagenomic data using deep learning. Quant Biol 2020;8:64–77. 34084563 10.1007/s40484-019-0187-4 PMC8172088

[btae192-B18] Romeo L , Frontoni E. A unified hierarchical XGBoost model for classifying priorities for COVID-19 vaccination campaign. Pattern Recognit 2022;121:108197. 34312570 10.1016/j.patcog.2021.108197 PMC8295058

[btae192-B19] Roux S , Faubladier M, Mahul A et al Metavir: a web server dedicated to virome analysis. Bioinformatics 2011;27:3074–5. 21911332 10.1093/bioinformatics/btr519

[btae192-B20] Sokolova M , Lapalme G. A systematic analysis of performance measures for classification tasks. Inf Process Manag 2009;45:427–37.

[btae192-B21] Song K , Ren J, Reinert G et al New developments of alignment-free sequence comparison: measures, statistics and next-generation sequencing. Brief Bioinform 2014;15:343–53. 24064230 10.1093/bib/bbt067 PMC4017329

[btae192-B22] Tampuu A , Bzhalava Z, Dillner J et al ViraMiner: deep learning on raw DNA sequences for identifying viral genomes in human samples. PLoS One 2019;14:e0222271. 31509583 10.1371/journal.pone.0222271 PMC6738585

[btae192-B23] Vinga S , Almeida J. Alignment-free sequence comparison-a review. Bioinformatics 2003;19:513–23. 12611807 10.1093/bioinformatics/btg005

[btae192-B24] Yu F , Wei C, Deng P et al Deep exploration of random Forest model boosts the interpretability of machine learning studies of complicated immune responses and lung burden of nanoparticles. Sci Adv 2021;7:eabf4130. 34039604 10.1126/sciadv.abf4130 PMC8153727

