# Supplementary material for: VirusPredictor: XGBoost-based software to predict virus-related sequences in human data
Source: Bioinformatics. 2024 Apr 10;40(4):btae192. doi: 10.1093/bioinformatics/btae192 (PMC11052659; doi:10.1093/bioinformatics/btae192)
Supplement: btae192_Supplementary_Data [file btae192_supplementary_data.zip › btae192_Supplementary_Data/VirusPredictor Supplementary Methods Tables and Figures.pdf]

Supplementary Methods, Tables, and Figures

Contents

Contents …………………………………………………………….…… Page 1

Supplementary Methods ………………………………………………... Pages 2 - 11

Supplementary Tables ……………………………………………….. Pages 12 - 36

Supplementary Figures ………………………………………………. Pages 37 - 38

Data and Software Availability ………………………………….... Page 39

References …………………………………………………..……… Pages 40 - 42

Supplementary Methods

S1 Supplementary Methods

The three subsections below are elaborated according to the order illustrated in **Figure 1**, including feature extraction and selection, our virus prediction XGBoost model to classify the three groups (infectious virus, human ERV, and non-ERV human), our subgroup prediction XGBoost model to classify six viral taxonomy groups, and our model assessment.

S1.1 Alignment-free feature extraction

To classify infectious virus, human ERV, and non-ERV human sequences, in this study we first employed feature extraction procedures, including 1) DNA sequence transformation into numerical sequences, and 2) informative numerical feature extraction (**Figure S1)**. Recently, the digital signal process DNA spectral analysis (Ahmad, et al., 2017; Sun, et al., 2023) has obtained success in classifying protein coding and noncoding regions. In this study, we used the same analysis to extract the features needed to distinguish infectious virus sequences from human ERV and non-ERV human sequences. Once the informative features were selected from the DNA sequences, we examined multiple machine learning classification approaches. We name our methods as **alignment-free feature extraction** in contrast to conventional alignment methods, such as BLASTN (Altschul, et al., 1990).

S1.1.1 DNA sequence transformation into numerical sequences

The K-tuple (or K-mer or K-word) method and recoding system were used to transfer DNA sequences into numerical sequences. The recoding system is comprised of three subgroup approaches: fixed mapping, physic-chemical property, and DNA-graph based long range correlation (**Figure S1** Step 1; **Figure S2**; and **Table S3**) (Akhtar, et al., 2007; Anastassiou, 2001; Buldyrev, et al., 1998; Chakravarthy, et al., 2004; Cristea, 2002; Florquin, et al., 2005; Hamori and Ruskin, 1983; Holden, et al., 2007; Jeffrey, 1990; Kwan, et al., 2012; Liu and Luan, 2014; Nair and Sreenadhan, 2006; Voss, 1992; Zhang, et al., 2003; Zhang, 2009). These approaches are described below (we use K-tuple, K-mer, and K-word interchangeably).

I. K-tuple method

K-tuple denotes a contiguous sequence of DNA bases that is K bases long (K=1,2…) (Ning, et al., 2001), for example, K=1: A, C, G, T ; K=2: AA, AC, AG, AT, …, TT ; K=3:AAA,AAC,ACA,…,TTT.

K-tuple (K-mer) size selection

All the possible K-mers' frequencies are calculated as potential features, where $K \leq p$, and the positive integer p can be calculated according to the literature (Akhter, et al., 2013). The details are as follows, K-mer size is selected according to formula (1),

$$n^p < \min{(S_i)}, \tag{1}$$

where $n = 4$, because there are a total of four nucleotides A, C, G, T. $K = 1,2, \ldots, p,\ i = 1,.., N$. S_i is the ith sequence length, N is sequence number in the database. If the minimum sequence length of a database is 100 bp, then p=3, thus, K=p=3.

The rationale of formula (1) is that a large K-mer size will produce erroneous and artificial differences between genomes that ultimately correlate with genome lengths rather than genome content (Akhter, et al., 2013; Borozan, et al., 2015). In this study, the minimum sequence length is 100 bp, so K=1, 2, 3, and we obtain $4^1 + 4^2 + 4^3$=84 K-mers. GC content is another important metric and thus added to the 84 K-mers. Thus, a total of four methods (i.e., K=1, 2, and 3 and GC content), and 85 features were obtained through the first DNA sequences transform group.

II. Recoding systems

A total of three types of methods were utilized to recode the sequences, including 1) fixed mapping, 2) physic-chemical property, and 3) DNA-graph based long range correlation. Each of the three types has multiple popular specific recoding methods, and a total of 19 alignment-free methods are utilized to extract features. The formulas of the 19 methods are summarized in **Table S3**.

S1.1.2 Numerical feature extraction

I. Features from K-tuples

The relative abundance,

$$\rho_{GT} = \frac{f_{GT}}{f_G f_T}, \tag{2}$$

where f_G, f_T, f_{GT} are the frequency of nucleotide G, T, and dinucleotide GT in a fixed length DNA sequence, respectively. When K=3, the relative abundance

$$\rho_{GTC} = \frac{f_{GTC}}{f_G f_T f_C}, \tag{3}$$

where f_G, f_T, f_C and f_{GTC} are the frequency of nucleotide G, T, C, and trinucleotide GTC in a fixed length DNA sequence, respectively. As for a potential future larger database, the features number in this model will adaptively change according to the database, i.e., $min\ (S_i)$, which will help improve the final prediction accuracy. In total, we selected 85 features (including GC content) through the K-tuple approach.

II. Features from recoding systems

According to the numerical sequence characteristics, i.e., whether they are real numbers (i.e., results of No. 1-4, 9-14, and 17-19 recoding methods in **Table S3**) or complex numbers (i.e., results of No. 5-8 and 15-16 recoding methods in **Table S3**), different approaches such as mathematical methods (Hoang, et al., 2015) and genomic signal processing methods (Abo-Zahhad, et al., 2012; Kwan, et al., 2012) were used to extract features.

III. Real numerical sequences

VOSS (Tiwari, et al., 1997; Voss, 1992) is the most popular numerical representation method in spectral analysis because of its perfect characteristics (Hoang, et al., 2015). Based on VOSS transformation of sequences, mathematical moments (Hoang) can be utilized to extract 1-order, 2-order and 3-order moments. We thus obtained 12 features from these analyses. For all the real numerical sequences, their statistical metrics such as mean, standard variance, skewness and kurtosis can be calculated. We thus obtained 88 features from these analyses. For all of the real numerical sequences except the VOSS transformed sequences, their Hurst exponent can also be calculated (Aste, 2013; Hamori and Ruskin, 1983). We thus obtained 24 features from these analyses.

IV. Complex numerical sequences

We calculated the Fourier coefficients' square of all the numerical sequences including the real and complex numerical sequences after fast Fourier transform (Tiwari, et al., 1997), and then calculated the mean values of the power spectra. After these procedures, we obtained 28 features.

A total of 152 features were extracted from the recoding system approaches. And after all the procedures, a DNA sequence of any length was represented by a numerical sequence with fixed length 85+152=237. Thus, if there are N DNA sequences in the input sequence data, an N × 237 numerical feature matrix will be generated after feature extraction, which is denoted as "original" whole feature matrix. Each row of the feature matrix represents the original DNA sequence (sample), and each column represents one of their 237 features. Loading this numerical feature matrix, our two-step XGBoost models are then trained to classify the three classes and the six classes as aforementioned.

S1.1.3 Feature selection

Once the original feature matrix is prepared, it is important to select informative features, also known as variable selection or feature engineering, i.e., the process of selecting a subset of informative features for model construction. The advantages of feature selection lie in improving the prediction performance of the predictors, providing faster and more cost-effective predictors, and providing a better understanding of the underlying process that generates the data (Guyon and Elisseeff, 2003). Feature selection plays a critical role in machine learning classification models. To perform the feature selection preprocess, we conducted the following procedures:

1) We randomly selected 302,169 samples from the whole three-class virus prediction training dataset (**Table S1**). 2) The well-known robust random forest (Fernández-Delgado, et al., 2014) model was utilized to train the 302,169 samples. During the training stage, we performed hyperparameter tuning via grid-search combined with three-fold cross validation to determine the optimal hyperparameters of the random forest model. 3) We obtained the feature importance with well-sorted order through leveraging the optimal random forest model (Yu, et al., 2021) (**Table S4**). 4) Based on the sorted importance features (**Table S4**), we extracted the top 20, 50, 100 features (i.e., columns) from the 302,169 training sequences described in step 1, and thus obtained three low-dimension training datasets. We obtained four different dimension training

datasets, and each of the four datasets had 302,169 samples (columns), while having 20, 50, 100, and all 237 features (rows), respectively. 5) Each of the four classification algorithms (i.e., random forest, K nearest neighbor, support vector machine, and XGBoost) were then trained on all the above four training datasets. All the machine learning approaches adopted the hyperparameter-tuning procedures by grid-search and three-fold cross validation to determine the optimal models. We used four performance metrics, including macro average accuracy, recall, precision, and F1-score (definition described in **Supplementary Methods S1.3**) to evaluate the four algorithms.

S1.2 Two-step XGBoost models for classifications

XGBoost (Chen and Guestrin, 2016) is an algorithm based on the integrated idea of boosting, which is improved on the basis of the algorithm of gradient boosting decision tree (GBDT), and uses classification and regression trees (CART) for classification and regression. Compared with the traditional GBDT algorithm, XGBoost has more advantages, such as: 1) it adds a regular term to the objective function and column sampling procedures to control the model complexity to overcome overfitting; 2) it utilizes second-order Taylor expansion on the loss function to improve accuracy of the model; 3) it can leverage the approximate algorithm to improve the greedy algorithm, and use multiple CPUs for parallel training at the same time, which improves the speed and can reach the optimum faster. In this study, we developed two XGBoost models, i.e., the virus prediction (infectious virus, human ERV, and non-ERV human) and subgroup prediction (dsDNA, ssDNA, Retro, ssRNA(-), ssRNA(+), and dsRNA) models.

S1.2.1 XGBoost procedures

The procedures of XGBoost are described below (Chen and Guestrin, 2016).

Step 1: Determine the model

Data $\mathrm{D} = \{(x_i, y_i)\}$ $(|\mathrm{D}| = n, x_i \in R^m, \ y_i \in R)$ has n samples and m features.

Model: Suppose there are k decision trees

$$\hat{y}_i = \Phi(x_i) = \sum_{k=1}^{K} f_k(x_i), \ f_k \in \mathrm{F} \tag{4}$$

where F $=\{f(x)=w_{q(x)}\}$ $(q: R^m \to T, w \in R^T)$ is CART's map space, w represents the weight vector of the leaf node, q is structure of the tree, T is the number of the leaf node.

Step 2: Determine the objective function

Objective function:

$$\mathrm{L}(\Phi) = \sum_i l(\hat{y}_i, y_i) + \sum_k \Omega(f_k), \tag{5}$$

where $\Omega(f_k) = \gamma T + \frac{1}{2}\|W\|^2$.

The objective function consists of two parts. The first part is the loss function, which measures how well the model fits the data, and the second part is the regular term, which controls the complexity of the model by penalizing the complexity of each tree, reduces the variance of the model, and prevents the data from overfitting. In formula (5), T in the regular term $\Omega(f_k)$ represents the number of leaf node, and w represents the score of leaf node.

Step 3: Perform additional training

Additional training is performed to find regularities to simplify the objective function

$$\hat{y}_i^{(t)} = \sum_{k=1}^{t} f_k(x_i) = \hat{y}_i^{(t-1)} + f_t(x_i), \tag{6}$$

where the $\hat{y}_i^{(t)}$ obtained by the *t*-th round of the decision tree is the sum of all the previous predicted values and the *t*-th predicted value.

Step 4: Optimize the objective function

(a) Rewrite the objective function

$$\mathrm{L}^{(t)} = \sum_{i=1}^{n} l(y_i, \hat{y}_i^{(t-1)} + f_t(x_i)) + \Omega(f_t) + cons\tan t. \tag{7}$$

(b) Use Taylor formula to rewrite the objective function

$$\mathrm{L}^{(t)} = \sum_{i=1}^{n} \left[g_i f_t(x_i) + \frac{1}{2} h_i f_t^2(x_i) \right] + \Omega(f_t), \tag{8}$$

where $g_i = \partial_{\hat{y}^{(t-1)}} l(y_i, \hat{y}_i^{(t-1)})$, $h_i = \partial^2_{\hat{y}^{(t-1)}} l(y_i, \hat{y}_i^{(t-1)})$.

(c) Redefine the tree and simplify the objective function

Use the *j*-th leaf set to define $I_j = \{i | q(x_i) = j\}$, and use w_i replace $f_t(x_i)$, we obtain the objective function:

$$L^{(t)} = \sum_{j=1}^{T} \left[(\sum_{i \in I_j} g_i) w_i + \frac{1}{2} (\sum_{i \in I_j} h_i + \lambda) w_i^2 \right] + \gamma T. \tag{9}$$

(d) Take the derivative of the objective function (9) with respect to w_i

We calculate the optimal weight w_j^* of leaf *j* by $w_j^* = -\frac{\sum_{i \in I_j} g_i}{\sum_{i \in I_j} h_i + \lambda}$, thus we obtain the corresponding optimal value by

$$L^{(t)}(q) = -\frac{1}{2} \sum_{j=1}^{T} \frac{(\sum_{i \in I_j} g_i)^2}{\sum_{i \in I_j} h_i + \lambda} + \gamma T. \tag{10}$$

The value of the objective function $L^{(t)}(q)$ is used to measure the quality of the *t*-th tree structure, which has nothing to do with *w*, and can be used as a standard to determine the best tree structure.

S1.2.2 Hyperparameter tuning

We optimized our models' hyperparameters to determine the optimal two-step XGBoost models. Hyperparmeters are the model parameters that could not be obtained in model training stage but must be set primarily based on expert experience, which is often a regular procedure in many machine learning problems. We performed a grid search algorithm with stratified three-fold cross validation to obtain the optimal hyperparameters combination. The following procedures helped to determine the optimal model hyperparameters and reduced the odds of overfitting: (1) The depth of the tree (*max_depth*), which indicates the specific degree of the learning sample. When the value is larger, the model is more prone to overfitting; (2) The number of trees (*n_estimatores*), which indicates the number of decision trees, that is, the number of iterations during training; (3) Learning rate (*learning_rate*), when the value is small, the training model takes a relatively long time, which can improve the timeliness of model training to a certain

extent; (4) Sample sampling rate (*subsample*), which controls the proportion of random sampling for each tree. Reducing the value of this parameter will make the algorithm more conservative and avoid overfitting; (5) Punishment coefficient (*gamma*), which specifies the minimum loss function descent value required for node splitting. The larger the gamma value, the more conservative the algorithm; (6) Minimum leaf node sample weight (*min_child_weight*), when the value is set large, it can effectively avoid local special samples Learning. The optimal hyperparameters of our two-step XGBoost models are shown in **Table S10**

S1.2.3 Normalization of training and testing data

Before the training and testing data are fed into our XGBoost models, it is necessary to normalize these data. It is known that large data values in classification may cause problems (Fan, et al., 2008), such as 1) features in larger numeric ranges may dominate those in smaller ranges; and 2) optimization methods for training may take longer time. Thus, we performed normalization for both training and testing datasets, which included the following two steps:

Step 1: The training datasets were tested by the MinMaxScaler strategy, which helped transform features by scaling each feature to a given range. MinMaxScaler uses formula

$$x_j^* = \frac{x_{ij} - \min(x_{.i})}{\max(x_{.i}) - \min(x_{.i})} \quad i = 1,2,\ldots,237. \tag{11}$$

where $\min(x_{.i})$, $\max(x_{.i})$ were the minimum and maximum values of column (feature) i. This formula translates each feature individually so that it is in the given range, i.e., between 0 and 1. Then, all the $\min(x_{.i})$, $\max(x_{.i})$ obtained from the training dataset were used to normalize the testing or predicting (i.e., unknown sequences) datasets. That was, each column of the testing and predicting numerical datasets was tested by MinMaxScaler using formula (11), where all the $\min(x_{.i})$, $\max(x_{.i})$ were obtained from the training dataset. This is a widely used machine learning normalization method, which ensures that all the involved training and testing processes have identical data structure and distribution.

Step 2: In the testing dataset normalization stage, the $\min(x_{.i})$, $\max(x_{.i})$ ($i = 1,2,\ldots,237$) obtained from the training dataset in Step 1 were used to normalize the testing datasets. These procedures likely helped keep the identical distribution and data structure for both training and testing datasets, and further improved accuracy of the model prediction.

S1.3 Model performance assessment

In machine learning classification, performance metrics are critical in model assessment and classifier selection. For binary classification, 2×2 confusion matrix is efficient and widely-used (Fawcett, 2006; Sokolova and Lapalme, 2009) for classifier assessment. For example, given a classifier for virus-human sequence prediction, there are four possible outcomes, including: True Positive (TP): a viral sequence (defined as positive event) is classified as virus (positive). False Negative (FN): a viral sequence is classified as human (defined as negative event). True Negative (TN): a human sequence is classified as human. False Positive (FP): a human sequence is classified as virus. Based on the definitions, the following performance metrics can be calculated and used in model assessment:

$$\text{Accuracy (A)} = \frac{TP+TN}{TP+FP+FN+TN},$$

$$\text{Recall (R)/Sensitivity/True Positive Rate (TPR)} = \frac{TP}{TP+FN},$$

$$\text{Precision (P)/Positive Predictive Value (PPV)} = \frac{TP}{TP+FP},$$

$$\text{Specificity/True Negative Rate (TNR)} = \frac{TN}{TN+FP},$$

$$\text{False Positive Rate (FPR)} = \frac{FP}{TN+FP},$$

$$\text{F1-score (F1)} = \frac{2*P*R}{P+R},$$

where P and R represent precision and recall, respectively.

Accuracy represents the percentage of correctly predicted over all testing virus and human. Recall/Sensitivity/True Positive Rate (TPR) represents among all the true viral sequences how many are correctly classified as viruses. Precision/Positive Predictive Value (PPV) means among all the sequences that are predicted as virus how many are true virus. Specificity/True Negative Rate (TNR) represents among all the true non-virus (including human ERV and Non-ERV human) sequences how many are correctly classified as non-virus. Thus, recall and precision are a pair of metrics to evaluate the classifier ability for virus prediction. F1-score is a combined metric of precision and recall for overall assessment as accuracy (Fawcett, 2006).

For our two-step (including three-class and then six-class) model predictions, similar metrics exist for model assessment based on the confusion matrix (Sokolova and Lapalme, 2009). In this study, we first counted the index values of each class (those predicted correctly belonging to this class are recorded as positive, and those not belonging to this class are recorded as negative), and then calculated the arithmetic mean of all classes. We used macro average accuracy, recall, precision, and F1-score to assess our two-step models, with their definition as follows:

$$macro\_C = \frac{1}{n}\sum_{i=1}^{n} A_i,$$

$$macro\_R = \frac{1}{n}\sum_{i=1}^{n} R_i,$$

$$macro\_P = \frac{1}{n}\sum_{i=1}^{n} P_i,$$

$$macro\_F_1 = \frac{1}{n}\sum_{i=1}^{n} F_{1_i},$$

where *i* represents the *i*-th class. The four metrics are similar to those used in binary classification scenarios, with the only difference being that in multiple classification scenario when recall of class i is calculated, class i is positive event, while the other classes are all negative events. For all the aforementioned metrics, the higher values represent better classification performance.

Supplementary Tables

Table S1 Training and testing sequences used in our virus prediction XGBoost model

Group	# of training sequences	# of testing sequences[a]			Total
		150-350 bp	850-950 bp	2,000-5,000 bp	
Infectious virus	1,486,462	938	937	963	1,489,300
Human ERV	1,512,342	1,000	1,000	1,000	1,515,342
Non-ERV human	1,575,686[b]	1,000	1,000	1,000	1,578,686
Total	4,574,490	2,938	2,937	2,963	4,583,328

[a], Length ranges of our testing sequences.

[b], The total length of non-endogenous retrovirus (ERV) human sequences used in this study is 266,701,535 base pair (bp), accounting for ~8.9% of the whole human genome.

To keep training sequences as many as possible, we randomly selected 1,000 sequences as the testing data for each group and length (9,000 testing sequences in total). Some numbers were less than 1,000 because of subsequent quality controls in which the sequences with missing nucleotides "N" were removed.

Table S2 Training and testing sequences used in our subgroup prediction XGBoost model

Viral subgroups	# of training sequences	# of testing sequences[a]			Total
		150-350 bp	850-950 bp	2,000-5,000 bp	
dsDNA	205,652	494	495	495	207,136
ssDNA	204,014	496	490	500	205,500
Retro	121,177	329	296	434	122,236
ssRNA(-)	201,691	498	491	488	203,168
ssRNA(+)	200,391	498	496	486	201,871
dsRNA	212,525	477	443	481	213,926
Total	1,145,450	2,792	2,711	2,884	1,153,837

[a], Length ranges of our testing sequences.

To keep training sequences as many as possible, we randomly selected 500 sequences as the testing data for each subgroup and length (9,000 testing sequences in total). The final numbers were less than 500 because of subsequent quality controls in which the sequences with missing nucleotides "N" were removed.

Table S3 Alignment-free methods used in recoding system

No	Class	Abbreviation	Description	Representations / Examples	#numerical sequences[a]	#features[b]
1	Fixed mapping	VOSS (Voss, 1992)	/	$X_n = \begin{cases} 1, S[n] = X, \\ 0, S[n] \neq X. \end{cases}$<br>$X_n$ applies to any A_n, C_n, G_n, T_n.	4	32
2		Integer (Cristea, 2002)	Integer number	*A=2, C=1, G=3, T=0*	1	6
3		SGI (Kwan, et al., 2012)	Single Galois indicator	*A=0, C=1, G=3, T=2*	1	6
4		Real (Chakravarthy, et al., 2004)	Real number	*A=-1.5, C=0.5, G=-0.5, T=1.5*	1	6
5		Complex (Anastassiou, 2001)	Complex number	*A=1+j, C=-1+j, G=-1-j, T=1-j*	1	2
6		KTPC (Kwan, et al., 2012)	K-twin-pair code	*A=j, C=-1, G=-1, T=j*	1	2
7		KBPC1 (Kwan, et al., 2012)	K-bipolar-pair code 1	*A=j, C=-1, G=1 T=-j*	1	2
8		KQC1 (Kwan, et al., 2012)	K-quaternary code 1	*A=1, C=-1, G=-j, T=j*	1	2
9	Physic-chemical property	EIIP (Nair and Sreenadhan, 2006)	Electron-ion interaction potential	*A=0.126, C=0.134, G=0.0806, T=0.1335*	1	6
10		PNAN (Kwan, et al., 2012)	Paired nucleotide atomic number	*A=62, C=42, G=62, T=42*	1	6
11		AN (Holden, et al., 2007)	Atomic number	*A=70, C=58, G=78, T=66*	1	6
12		MM (Kwan, et al., 2012)	Molecular mass	*A=134, C=110, G=150, T=125*	1	6
13		PN (Akhtar, et al., 2007)	Paired numeric	*A=1, C=-1, G=-1, T=1*	1	6
14		SP (Florquin, et al., 2005)	Structure profile	DNA-bending stiffness: *AA=35, AC=60 ,…, TT=35*<br>Duplex free energy: *AA=-12, AC=-15 ,…, TT=35*<br>Duplex disrupt energy: *AA=19, AC=13 ,…, TT=19*<br>Propeller twist: *AA=-1886, AC=-1310 ,…, TT=-1866*	4	24

15		DNA walk (Buldyrev, et al., 1998)	/	*A or T=1, C or G=-1*	1	2
16	DNA-graph based long range correlation	Hcurve (Hamori and Ruskin, 1983)	/	*A=i+j-k, C=-i-j-k, G=-i+j-k, T=i+j-k*	1	2
17		CGR curve (Jeffrey, 1990)	Chaos game representation	$CGR_i = CGR_{i-1} - 0.5(CGR_{i-1} - g_i)$,<br>$\mathrm{i} = 1,2,3,\ldots,\mathrm{N};\ CGR_0 = (0.5,0.5)$;<br>$g_i = \{A(0,0), C(0,1), G(1,1), T(1,0)\}$	2	12
18		Dv curve (Zhang, 2009)	Dual-vector curve	$(x_0, y_0) = (0,0)$<br>$x_{2i-1} = 2\mathrm{i} - 1, \mathrm{i} = 1,2,\ldots,\mathrm{n}$<br>$x_{2i} = 2i, \mathrm{i} = 1,2,\ldots,\mathrm{n}$,<br>$y_{2i-1} = \begin{cases} y_{2i-2} + 1, if\ S_i = A\ or\ T \\ y_{2i-2} - 1, if\ S_i = C\ or\ G' \end{cases}$<br>$i = 1,2,..,n$<br>$y_{2i} = \begin{cases} y_{2i-1} + 1, if\ S_i = A\ or\ C \\ y_{2i-1} - 1, if\ S_i = T\ or\ G' \end{cases}$<br>$i = 1,2,..,n$	1	6
19		Zcurve (Zhang, et al., 2003)	/	$\begin{cases} x_n = (A_n + G_n) - (C_n + T_n) \\ y_n = (A_n + C_n) - (G_n + T_n) \\ z_n = (A_n + T_n) - (G_n + C_n) \end{cases}$<br>$x_n, y_n, z_n \in [-N, N], n = 0,1,2,\ldots,$	3	18

[a], Number of numerical sequences obtained for an input DNA sequence after the transformation using the corresponding recoding method.

[b], Number of features generated via the procedures as described in Supplementary Methods S1.1.2 subsections II, III, and IV.

Table S4 Features ranked by our random forest approach (Excel File)

Table S5 Performance metric comparisons among four machine learning algorithms using different number of features

Algorithm	# of Features[a]	Accuracy[b] (macro)	Recall[b] (macro)	Precision[b] (macro)	F1-score[b] (macro)
Random forest	Top 20	0.783	0.785	0.788	0.786
	Top 50	0.845	0.845	0.847	0.846
	Top 100	0.865	0.866	0.868	0.866
	All 237	0.851	0.851	0.854	0.853
K nearest neighbor	Top 20	0.73	0.732	0.732	0.731
	Top 50	0.802	0.802	0.806	0.804
	Top 100	0.801	0.802	0.805	0.803
	All 237	0.783	0.783	0.79	0.785
Support vector machine	Top 20	0.813	0.724	0.720	0.721
	Top 50	0.871	0.809	0.806	0.807
	Top 100	0.877	0.819	0.816	0.817
	All 237	0.893	0.842	0.839	0.840
XGBoost	Top 20	0.787	0.788	0.789	0.788
	Top 50	0.874	0.874	0.875	0.874
	Top 100	0.895	0.895	0.896	0.896
	All 237	0.904	0.904	0.904	0.904

[a], Number of features tested, i.e., top 20, 50 or 100 most important features or all 237 features.

[b], Only subset sequences (i.e., 302,169 sequences randomly selected from our whole 4,583,328 sequences) were used to examine all the four machine learning algorithms because of the computational constraints of our computing cluster. We selected 15,108 (accounting for 5%) as the testing sequences, with the rest as the training sequences.

The macro average accuracy, recall, precision, and F1-score were calculated according to the definitions described in Supplementary Methods S1.3.

Table S6 Performance summary of our virus prediction XGBoost model

Length of sequences (bp)	Accuracy (macro)	Recall (macro)	Precision (macro)	F1-score (macro)
2,000-5,000	0.978	0.968	0.967	0.967
850-950	0.930	0.902	0.896	0.892
150-350	0.760	0.756	0.643	0.595

Table S7 Pair-wise confusion matrix of our virus prediction XGBoost model

Table S7A Pair-wise confusion matrix

2,000-5,000 bp

		Predicted			Total[b]
		Infectious virus[a]	Human ERV[a]	Non-ERV human[a]	
True	Non-ERV human	33	1	966	1,000
	Human ERV	30	947	23	1,000
	Infectious virus	952	2	9	963

850-950 bp

		Predicted			Total
		Infectious virus	Human ERV	Non-ERV human	
True	Non-ERV human	110	161	729	1,000
	Human ERV	7	979	14	1,000
	Infectious virus	919	7	11	937

150-350 bp

		Predicted			Total
		Infectious virus	Human ERV	Non-ERV human	
True	Non-ERV human	157	652	191	1,000
	Human ERV	14	975	11	1,000
	Infectious virus	715	220	3	938

[a], Predicted number.

[b], The total number of testing sequences.

The numbers on the diagonal represent correct predictions.

In this study, we used a small portion (~8.9%) of the non-ERV human genome sequences (randomly selected) as the training data. This was because it is currently not possible to use the whole genome sequence due to the limitations of current CPU/GPU power. As soon as more powerful computers become available, as a larger portion or the complete non-ERV human sequences are included in the training process, the accuracies to predict the non-ERV human group can increase significantly (our software has provided the codes which are readily available for use).

Table S7B Model performance metrics

Length	2,000-5,000 bp			850-950 bp			150-350 bp		
Measures	Virus vs. Non-virus	ERV vs. Non-ERV	Non-ERV human vs. Virus + ERV	Virus vs. Non-virus	ERV vs. Non-ERV	Non-ERV human vs. Virus + ERV	Virus vs. Non-virus	ERV vs. Non-ERV	Non-ERV human vs. Virus + ERV
Accuracy	0.975	0.967	0.967	0.954	0.895	0.895	0.866	0.64	0.64
Precision/Positive predictive value	0.938	0.955	0.938	0.887	0.772	0.949	0.807	0.486	0.435
Sensitivity/Recall/True positive rate	0.989	0.947	0.966	0.981	0.979	0.729	0.762	0.975	0.191
Specificity/True negative rate	0.969	0.977	0.967	0.942	0.851	0.98	0.915	0.468	0.872
False positive rate	0.032	0.023	0.033	0.059	0.149	0.02	0.086	0.533	0.128
F1 score	0.963	0.951	0.952	0.932	0.863	0.825	0.784	0.648	0.265

Note: The definition and conversion approaches of binary classes (e.g., virus vs. non-virus) are shown in **Table S11B**.

Table S8 Performance of our subgroup prediction XGBoost model

Length of sequences (bp)	Accuracy (macro)	Recall (macro)	Precision (macro)	F1-score (macro)
2,000-5,000	0.983	0.957	0.947	0.950
850-950	0.981	0.953	0.939	0.945
150-350	0.919	0.849	0.653	0.727

Table S9 Pair-wise confusion matrix of our subgroup prediction XGBoost model

2,000-5,000 bp

		Predicted								
		dsDNA[a]	dsRNA[a]	Retro[a]	ssDNA[a]	ssRNA(-)[a]	ssRNA(+)[a]	Human ERV[a]	Non-ERV human[a]	Total[b]
True	ssRNA(+)	5	1	0	0	2	468	0	5	481
	ssRNA(-)	5	0	0	0	473	0	0	8	486
	ssDNA	24	0	0	464	0	0	0	0	488
	Retro	2	0	425	0	0	1	2	4	434
	dsRNA	5	411	0	84	0	0	0	0	500
	dsDNA	487	7	0	0	0	0	0	1	495

850-950 bp

		Predicted								
		dsDNA	dsRNA	Retro	ssDNA	ssRNA(-)	ssRNA(+)	Human ERV	Non-ERV human	Total
True	ssRNA(+)	3	4	0	4	3	422	3	4	443
	ssRNA(-)	2	1	1	0	474	4	12	2	496
	ssDNA	6	3	0	468	2	12	0	0	491
	Retro	1	0	285	1	3	2	4	0	296
	dsRNA	9	465	0	2	1	13	0	0	490
	dsDNA	426	15	0	9	6	26	3	10	495

150-350 bp

		Predicted								
		dsDNA	dsRNA	Retro	ssDNA	ssRNA(-)	ssRNA(+)	Human ERV	Non-ERV human	Total
True	ssRNA(+)	12	12	0	6	11	361	72	3	477
	ssRNA(-)	8	9	3	2	282	33	158	3	498
	ssDNA	16	12	1	354	1	45	64	5	498
	Retro	0	0	175	0	3	7	131	13	329
	dsRNA	22	390	0	8	6	37	30	3	496
	dsDNA	281	42	1	24	4	81	52	9	494

[a], Predicted number.

[b], Total number of testing sequences.

The numbers on the diagonal represent correct predictions (except columns Human ERV and Non-ERV human).

ERVs are derived from ancient retro-transcribing viral infections and integrations into the human genome. Thus, ERVs share high sequence similarities with modern retro-transcribing viruses and other single-stranded RNA viruses, indicated by our observation. This study is the first to consider ERVs in infectious viral sequence classification, and our results suggest that ERVs should be considered in all future similar research.

Table S10 Optimal hyperparameters of our trained two-step XGBoost models

Parameters	Range	Optimal value of virus prediction model	Optimal value of subgroup prediction model
max_depth[a]	[4,6,8]	8	8
n_estimatores[b]	200	200	200
learning_rate[c]	[0.05, 0.1,0.2]	0.2	0.2
subsample[d]	[0.5,0.8]	0.8	0.8
gamma[e]	0	0	0
min_child_weight[f]	[0,2,4]	0	0

[a], *max_depth*: Maximum tree depth for base learners. Increasing this value will make the model more complex and more likely to overfit.

[b], *n_estimators*: Number of gradient boosted trees; Equivalent to number of boosting rounds.

[c], *learning_rate*: Step size shrinkage used in update to prevent overfitting.

[d], *subsample*: Subsample ratio of the training instances. This will prevent overfitting.

[e], *gamma*: Minimum loss reduction required to make a further partition on a leaf node of the tree. The larger gamma is, the more conservative the algorithm will be.

[f], *min_child_weight*: Minimum sum of instance weight (hessian) needed in a child. If the tree partition step results in a leaf node with the sum of instance weight less than min_child_weight, the building process will give up further partitioning.

Additional sources: https://xgboost.readthedocs.io/en/stable/parameter.html; and https://xgboost.readthedocs.io/en/stable/python/python_api.html.

Table S11 Pair-wise confusion matrix and model performance metrics of our virus prediction XGBoost model to predict 3,761 viral contigs

Table S11A Pair-wise confusion matrix

		Predicted			
		Infectious virus[a]	Human ERV[a]	Non-ERV human[a]	Total[b]
True	Non-ERV human	18	4	3,978	4,000
	Human ERV	7	3,869	124	4,000
	Infectious virus	3,747	0	14	3,761

[a], Predicted number.

[b], The total number of testing sequences.

The 3,761 contigs were from the study (Kowarsky, et al., 2017). The 4,000 ERV and 4,000 non-ERV human sequences were randomly selected from the whole genome, i.e., the entire ERV and non-ERV human sequences, respectively.

No sub-group prediction was conducted because of no virus subgroup taxonomy information available in this dataset.

Table S11B Model performance metrics

Measures	Virus vs. Non-virus	ERV vs. Non-ERV	Non-ERV human vs. Virus + ERV
Accuracy	0.997	0.986	0.986
Precision	0.993	0.991	0.965
Sensitivity	0.996	0.967	0.995
Specificity	0.997	0.995	0.981
False positive rate	0.003	0.005	0.019
F1 score	0.995	0.979	0.979

The tables below show the pair-wise confusion matrix that we used to compute the above performance metrics. For example, to convert three classes to binary classes, we combined ERV and non-ERV human as non-virus to obtain the virus vs. non-virus binary class.

Virus vs. Non-virus

Predicted

		Infectious virus	Non-virus	Total
	Non-virus (negative)	25	7,975	8,000
True	Infectious virus (positive)	3,747	14	3,761

ERV vs. Non-ERV

		Predicted		
		Human ERV	Non-human ERV	Total
	Non-human ERV (negative)	36	7,725	7,761
True	Human ERV (positive)	3,869	131	4,000

Non-ERV human vs. Virus + ERV

		Predicted		
		Non-ERV human	Virus + ERV	Total
	Virus + ERV (negative)	145	7,616	7,761
True	Non-ERV human (positive)	3,978	22	4,000

Table S12 Prediction results of SARS-CoV-2 fragments (Excel File)

Table S13 Pair-wise confusion matrix and model performance metrics of our virus prediction XGBoost model to predict SARS-CoV-2 sequences

Table S13A Pair-wise confusion matrix

2,000-5,000 bp

		Predicted			Total[b]
		Infectious virus[a]	Human ERV[a]	Non-ERV human[a]	
True	Non-ERV human	0	0	100	100
	Human ERV	0	97	3	100
	Infectious virus	100	0	0	100

850-950 bp

		Predicted			Total[b]
		Infectious virus[a]	Human ERV[a]	Non-ERV human[a]	
True	Non-ERV human	2	8	90	100
	Human ERV	0	96	4	100
	Infectious virus	97	0	3	100

150-350 bp

		Predicted			Total[b]
		Infectious virus[a]	Human ERV[a]	Non-ERV human[a]	
True	Non-ERV human	6	74	20	100
	Human ERV	0	99	1	100
	Infectious virus	69	31	0	100

[a], Predicted number.

[b], The total number of testing sequences.

The 100 ERV and 100 non-ERV human sequences in each length group were randomly selected from the whole genome, i.e., the entire ERV and non-ERV human sequences, respectively.

Table S13B Model performance metrics

Length	2,000-5,000 bp			850-950 bp			150-350 bp		
Measures	Virus vs. Non-virus	ERV vs. Non-ERV	Non-ERV human vs. Virus + ERV	Virus vs. Non-virus	ERV vs. Non-ERV	Non-ERV human vs. Virus + ERV	Virus vs. Non-virus	ERV vs. Non-ERV	Non-ERV human vs. Virus + ERV
Accuracy	1	0.99	0.99	0.983	0.943	0.943	0.877	0.627	0.627
Precision	1	1	0.971	0.98	0.881	0.928	0.92	0.471	0.385
Sensitivity	1	0.97	1	0.97	0.96	0.9	0.69	0.99	0.2
Specificity	1	1	0.985	0.99	0.935	0.965	0.97	0.445	0.84
False positive rate	0	0	0.015	0.01	0.065	0.035	0.03	0.555	0.16
F1 score	1	0.985	0.985	0.975	0.919	0.914	0.789	0.639	0.263

Table S14 Pair-wise confusion matrix and model performance metrics of our virus prediction XGBoost model to predict Illumina 300 bp virus reads of human metagenomic data

Table S14A Pair-wise confusion matrix

		Predicted			
		Infectious virus[a]	Human ERV[a]	Non-ERV human[a]	Total[b]
True	Non-ERV human	305	725	4,570	5,600
	Human ERV	176	4,515	909	5,600
	Infectious virus	4,622	159	770	5,551

[a], Predicted number.

[b], The total number of testing sequences.

The 5,600 ERV and 5,600 non-ERV human sequences were randomly selected from the whole genome, i.e., the entire ERV and non-ERV human sequences, respectively.

No sub-group prediction was conducted because of no virus subgroup taxonomy information available in this dataset.

Table S14B Model performance metrics

Measures	Virus vs. Non-virus	ERV vs. Non-ERV	Non-ERV human vs. Virus + ERV
Accuracy	0.916	0.818	0.818
Precision	0.906	0.697	0.694
Sensitivity	0.833	0.806	0.816
Specificity	0.957	0.824	0.819
False positive rate	0.043	0.176	0.181
F1 score	0.868	0.748	0.75

Table S15 Pair-wise confusion matrix and model performance metrics of our virus prediction XGBoost model to predict phage reference sequences

Table S15A Pair-wise confusion matrix

3,000 bp

		Predicted			Total[b]
		Infectious virus[a]	Human ERV[a]	Non-ERV human[a]	
True	Non-ERV human	1	3	996	1,000
	Human ERV	0	996	4	1,000
	Infectious virus	993	0	0	993

1,000 bp

		Predicted			Total[b]
		Infectious virus[a]	Human ERV[a]	Non-ERV human[a]	
True	Non-ERV human	12	48	940	1,000
	Human ERV	6	932	62	1,000
	Infectious virus	995	0	0	995

500 bp

		Predicted			Total[b]
		Infectious virus[a]	Human ERV[a]	Non-ERV human[a]	
True	Non-ERV human	25	101	874	1,000
	Human ERV	23	857	120	1,000
	Infectious virus	988	1	9	998

300 bp

		Predicted			Total[b]
		Infectious virus[a]	Human ERV[a]	Non-ERV human[a]	
True	Non-ERV human	51	121	828	1,000
	Human ERV	35	815	150	1,000
	Infectious virus	976	1	20	997

150 bp

		Predicted			Total[b]
		Infectious virus[a]	Human ERV[a]	Non-ERV human[a]	
True	Non-ERV human	97	158	745	1,000
	Human ERV	57	717	226	1,000
	Infectious virus	934	11	54	999

[a], Predicted number.

[b], The total number of testing sequences. The numbers of infectious viruses were less than 1,000 because of subsequent quality controls in which the sequences with missing nucleotides "N" were removed.

The 1,000 ERV and 1,000 non-ERV human sequences in each length group were randomly selected from the whole genome, i.e., the entire ERV and non-ERV human sequences, respectively.

Note: The study (Ren, et al., 2020) also compared lengths of sequences. It used 752 viral reference genomes as testing data. We found that 35 of the 752 genomes no longer exist in the NCBI viral reference genome database. After removing sequences with missing nucleotides, we eventually obtained 674 genomes for our analyses.

Table S15B Model performance metrics

Length	3,000 bp			1,000 bp			500 bp			300 bp			150 bp		
Measures	Virus vs. Non-virus	ERV vs. Non-ERV	Non-ERV human vs. Virus + ERV	Virus vs. Non-virus	ERV vs. Non-ERV	Non-ERV human vs. Virus + ERV	Virus vs. Non-virus	ERV vs. Non-ERV	Non-ERV human vs. Virus + ERV	Virus vs. Non-virus	ERV vs. Non-ERV	Non-ERV human vs. Virus + ERV	Virus vs. Non-virus	ERV vs. Non-ERV	Non-ERV human vs. Virus + ERV
Accuracy	1	0.997	0.997	0.994	0.957	0.957	0.981	0.907	0.907	0.964	0.874	0.874	0.927	0.799	0.799
Precision	0.999	0.996	0.996	0.982	0.940	0.933	0.954	0.863	0.851	0.919	0.809	0.801	0.858	0.691	0.682
Sensitivity	1	0.996	0.996	1	0.932	0.940	0.990	0.857	0.874	0.979	0.815	0.828	0.935	0.717	0.745
Specificity	1	0.998	0.998	0.991	0.970	0.966	0.976	0.932	0.923	0.957	0.903	0.897	0.923	0.840	0.826
False positive rate	0.001	0.002	0.002	0.009	0.030	0.034	0.024	0.068	0.077	0.043	0.097	0.103	0.077	0.160	0.174
F1 score	0.999	0.996	0.996	0.991	0.936	0.936	0.971	0.860	0.862	0.948	0.812	0.814	0.895	0.704	0.712

Table S16 Pair-wise confusion matrix and model performance metrics of our virus prediction XGBoost model to predict DNA virus sequences from human gut microbiome

Table S16A Pair-wise confusion matrix

30,000-100,000 bp

		Predicted			Total[b]
		Infectious virus[a]	Human ERV[a]	Non-ERV human[a]	
True	Non-ERV human	42	0	2,458	2,500
	Human ERV	0	2,500	0	2,500
	Infectious virus	2,500	0	0	2,500

10,000-30,000 bp

		Predicted			Total[b]
		Infectious virus[a]	Human ERV[a]	Non-ERV human[a]	
True	Non-ERV human	1	0	2,499	2,500
	Human ERV	0	2,500	0	2,500
	Infectious virus	2,499	0	1	2,500

2,000-5,000 bp

		Predicted			Total[b]
		Infectious virus[a]	Human ERV[a]	Non-ERV human[a]	
True	Non-ERV human	3	0	4,997	5,000
	Human ERV	2	4,833	165	5,000
	Infectious virus	4,979	0	10	4,989

850-950 bp

		Predicted			Total[b]
		Infectious virus[a]	Human ERV[a]	Non-ERV human[a]	
True	Non-ERV human	52	685	4,263	5,000
	Human ERV	26	4,859	115	5,000
	Infectious virus	4,954	10	26	4,990

150-350 bp

		Predicted			Total[b]
		Infectious virus[a]	Human ERV[a]	Non-ERV human[a]	
True	Non-ERV human	227	3,572	1,201	5,000
	Human ERV	49	4,882	69	5,000
	Infectious virus	4,558	402	28	4,988

[a], Predicted number.

[b], The total number of testing sequences.

The 5,000 ERV and 5,000 non-ERV human sequences in each length group were randomly selected from the whole genome, i.e., the entire ERV and non-ERV human sequences, respectively.

Table S16B Model performance metrics

Length	30,000-100,000 bp			10,000-30,000bp			2,000-5,000 bp			850-950 bp			150-350 bp		
Measures	Virus vs. Non-virus	ERV vs. Non-ERV	Non-ERV human vs. Virus + ERV	Virus vs. Non-virus	ERV vs. Non-ERV	Non-ERV human vs. Virus + ERV	Virus vs. Non-virus	ERV vs. Non-ERV	Non-ERV human vs. Virus + ERV	Virus vs. Non-virus	ERV vs. Non-ERV	Non-ERV human vs. Virus + ERV	Virus vs. Non-virus	ERV vs. Non-ERV	Non-ERV human vs. Virus + ERV
Accuracy	0.994	0.994	0.994	0.999	0.999	0.999	0.999	0.988	0.988	0.992	0.939	0.939	0.953	0.71	0.71
Precision	0.984	0.984	1	0.999	0.999	0.999	0.999	0.997	0.966	0.985	0.863	0.96	0.943	0.536	0.687
Sensitivity	1	1	0.983	0.999	1	0.999	0.998	0.967	0.999	0.993	0.972	0.853	0.914	0.976	0.24
Specificity	0.992	0.992	1	0.999	0.999	0.999	0.999	0.999	0.982	0.992	0.923	0.982	0.972	0.577	0.945
False positive rate	0.008	0.008	0	1	1	1	0.001	0.001	0.018	0.008	0.077	0.018	0.028	0.423	0.055
F1 score	0.992	0.992	0.992	1	1	1	0.998	0.982	0.982	0.989	0.914	0.903	0.928	0.692	0.356

Table S16C Virus group and subgroup predictions

			Virus prediction[c]		Virus subgroup prediction[d]	
Sequence lengths (bp)	No. sequences[a]	Post-QC sequences[b]	Correct prediction	Sensitivity	Correct prediction	Sensitivity
30,000-100,000	2,500	2,500	2,500	1	2,465	0.986
10,000-30,000	2,500	2,500	2,499	0.999	2,425	0.97
2,000-5,000	5,000	4,989	4,979	0.998	4,730	0.95
850-950	5,000	4,990	4,954	0.993	4,018	0.811
150-350	5,000	4,988	4,558	0.914	2,577	0.565

[a], Sequences randomly selected from the 189,680 virus sequences of the human gut microbiome.

[b], QC: quality control. Sequences with taxonomy label “NULL” were removed from the analyses.

[c], Virus prediction model to classify among infectious virus, ERV, and non-ERV human.

[d], Subgroup prediction model to classify among ssRNA(+), ssRNA(-), ssDNA, Retro, dsRNA, and dsDNA.

Supplementary Figures

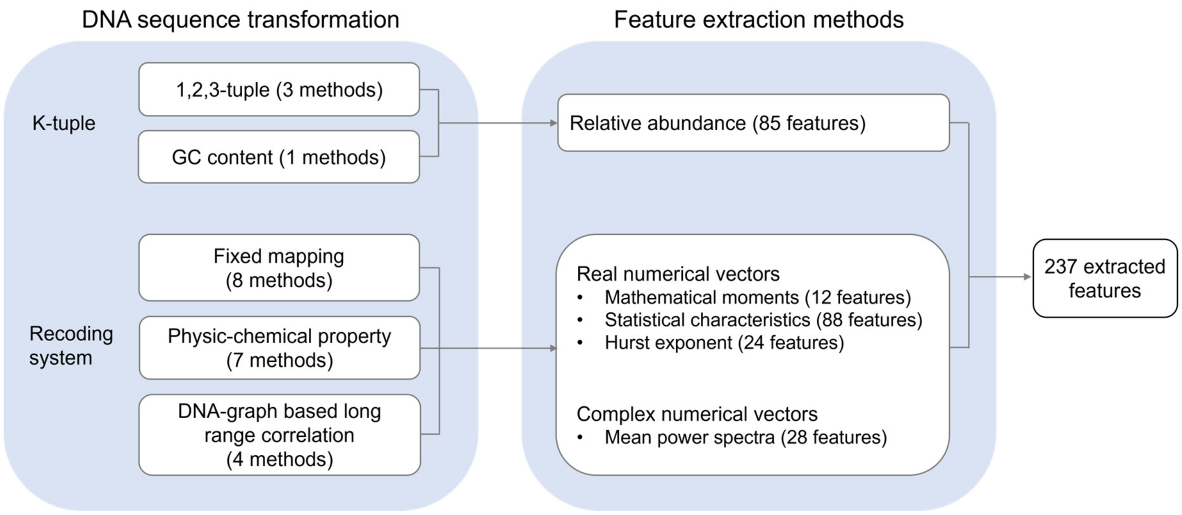

Figure S1 DNA sequence transformation and feature extraction strategy. Our alignment-free feature extraction process includes two steps. In the first step, two complementary mapping methods, i.e., K-tuple and recoding system, are used to transfer DNA sequences into numerical vectors. The K-tuple strategy includes tuple methods and GC content method (four methods in total). The recoding system includes three sub-recoding systems, i.e., fixed mapping (which includes 8 specific methods), physic-chemical property (7 methods), and DNA-graph based long range correlation (4 methods). Thus, a total of 23 methods are involved in the whole sequence transformation process. In the second step, two feature extraction methods are utilized on the numerical vectors generated from step 1, i.e., for the numerical vectors generated from K-tuple method, the relative abundance approach is used, and 85 features are obtained including features from GC content. For the numerical vectors generated from the recoding system, four specific feature extraction approaches are used according to the property of the numerical vectors. Specifically, for real numerical vectors, three methods are used, including a) mathematical moments based on FFT for VOSS (12 features generated), b) four statistical characteristics, i.e., mean, standard variance, skewness, kurtosis (88 features), and c) Hurst exponent (24 features). For complex numerical vectors, combing all the above real numerical vectors, the FFT-based mean power spectra is utilized to extract features (28 features). According to these procedures, a total of 237 features are extracted from the input DNA sequences. **Supplementary Methods S1.1.2 subsection II, III**, and **IV**, and **Table S3** show the details.

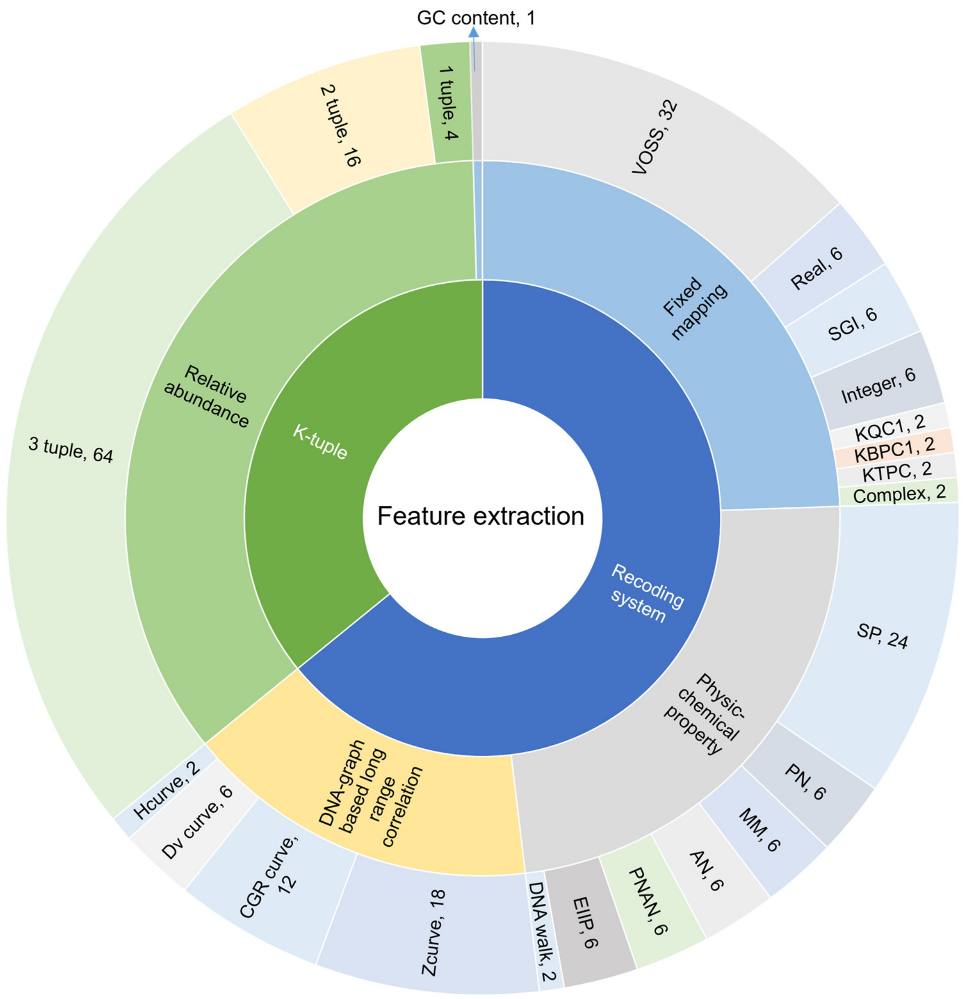

Figure S2 Sunburst chart of hierarchical structure of feature extraction methods. The procedures include two steps: 1) transforming DNA sequences to numerical sequences and 2) extracting features via different strategy. In the first step, two kinds of strategies, i.e., K-tuple and recoding system, are utilized to transform A, C, G, and T nucleotide DNA sequences to numerical sequences. In the K-tuple strategy, K=1,2,3 tuple partial DNA sequences are extracted, and the relative abundance features of the tuples are calculated; then GC content is calculated for the DNA sequence. Similarly, the recoding system strategy includes three subgroup strategies, i.e., fixed mapping, physic-chemical property, and DNA-graph based long range correlation. In each of the three subgroup strategies, different numbers of specific methods are shown in this figure. The details are shown in **Table S3**. The number after the method name represents the features extracted via this method, for example, "2 tuple, 16", where "2 tuple" is the feature extraction method, and 16 is the features extracted via the "2 tuple" method. The details are shown in **Supplementary Methods S1.1.2 subsection II, III**, and **IV**, **Figure S1**, and **Table S3**.

Data and software availability

URLs for data presented herein are as follows:

NCBI Human Genome Resources: www.ncbi.nlm.nih.gov/projects/genome/guide/human

Human genome reference GRCh38.p13: www.ncbi.nlm.nih.gov/assembly/GCF_000001405.39

Transposable element annotation hg38_rmsk_TE.gtf.gz: http://hammelllab.labsites.cshl.edu/software

NCBI Consensus Coding Sequence database: https://ftp.ncbi.nlm.nih.gov/pub/CCDS

NCBI Viral Genomes: www.ncbi.nlm.nih.gov/genome/viruses

NCBI Viral Genomes database: https://ftp.ncbi.nlm.nih.gov/refseq/release/viral

International Committee on Taxonomy of Viruses: https://talk.ictvonline.org/ictv-reports/ictv_online_report

Baltimore classification: http://viralzone.expasy.org/254

bedtools/getfasta: https://bedtools.readthedocs.io/en/latest/content/tools/getfasta.html

VirusPredictor Python package: www.dllab.org/software/VirusPredictor.html

VirusPredictor transposable element-masked human genome reference GRCh38.p13: www.dllab.org/software/VirusPredictor/hg38_rmsk_ERV.fa

VirusPredictor endogenous retrovirus training data (minimum length = 100 bp)**:** www.dllab.org/software/VirusPredictor/hg38_ERV_100bp.fa

References

Abo-Zahhad, M., Ahmed, S.M. and Abd-Elrahman, S.A. Genomic analysis and classification of exon and intron sequences using DNA numerical mapping techniques. *International Journal of Information Technology and Computer Science (IJITCS)* 2012;4(8):22.

Ahmad, M., Jung, L.T. and Bhuiyan, A.A. A biological inspired fuzzy adaptive window median filter (FAWMF) for enhancing DNA signal processing. *Comput Methods Programs Biomed* 2017;149:11-17.

Akhtar, M., Epps, J. and Ambikairajah, E. Paired spectral content measure for gene and exon prediction in eukaryotes. In, *Information and Emerging Technologies, 2007. ICIET 2007. International Conference on*. IEEE; 2007. p. 1-4.

Akhter, S., *et al.* Applying Shannon's information theory to bacterial and phage genomes and metagenomes. *Scientific reports* 2013;3.

Altschul, S.F., *et al.* Basic local alignment search tool. *Journal of molecular biology* 1990;215(3):403-410.

Anastassiou, D. Genomic signal processing. *Signal Processing Magazine, IEEE* 2001;18(4):8-20.

Aste, T. Generalized Hurst exponent (https://www.mathworks.com/matlabcentral/fileexchange/30076-generalized-hurst-exponent). 2013.

Borozan, I., Watt, S. and Ferretti, V. Integrating alignment-based and alignment-free sequence similarity measures for biological sequence classification. *Bioinformatics* 2015:btv006.

Buldyrev, S., *et al.* Analysis of DNA sequences using methods of statistical physics. *Physica A: Statistical Mechanics and its Applications* 1998;249(1):430-438.

Chakravarthy, N., *et al.* Autoregressive modeling and feature analysis of DNA sequences. *EURASIP Journal on Applied Signal Processing* 2004;2004:13-28.

Chen, T. and Guestrin, C. XGBoost: A Scalable Tree Boosting System. In, *Proceedings of the 22nd ACM SIGKDD International Conference on Knowledge Discovery and Data Mining*. San Francisco, California, USA: Association for Computing Machinery; 2016. p. 785–794.

Cristea, P.D. Genetic signal representation and analysis. In, *International Symposium on Biomedical Optics*. International Society for Optics and Photonics; 2002. p. 77-84.

Fan, R.-E., *et al.* LIBLINEAR: A library for large linear classification. *The Journal of Machine Learning Research* 2008;9:1871-1874.

Fawcett, T. An introduction to ROC analysis. *Pattern recognition letters* 2006;27(8):861-874.

Fernández-Delgado, M., *et al.* Do we need hundreds of classifiers to solve real world classification problems? *The Journal of Machine Learning Research* 2014;15(1):3133-3181.

Florquin, K., *et al.* Large-scale structural analysis of the core promoter in mammalian and plant genomes. *Nucleic acids research* 2005;33(13):4255-4264.

Guyon, I. and Elisseeff, A. An introduction to variable and feature selection. *The Journal of Machine Learning Research* 2003;3:1157-1182.

Hamori, E. and Ruskin, J. H curves, a novel method of representation of nucleotide series especially suited for long DNA sequences. *Journal of Biological Chemistry* 1983;258(2):1318-1327.

Hoang, T. Cluster DNA sequences using Fourier power spectrum (Version 1.0) (https://www.mathworks.com/matlabcentral/mlc-downloads/downloads/submissions/49026/versions/1/previews/GetMomentVectorPS.m/index.html).

Hoang, T., *et al.* A new method to cluster DNA sequences using Fourier power spectrum. *Journal of theoretical biology* 2015;372:135-145.

Holden, T., *et al.* ATCG nucleotide fluctuation of Deinococcus radiodurans radiation genes. In, *Optical Engineering+ Applications*. International Society for Optics and Photonics; 2007. p. 669417-669417-669410.

Jeffrey, H.J. Chaos game representation of gene structure. *Nucleic Acids Research* 1990;18(8):2163-2170.

Kowarsky, M., *et al.* Numerous uncharacterized and highly divergent microbes which colonize humans are revealed by circulating cell-free DNA. *Proc Natl Acad Sci U S A* 2017;114(36):9623-9628.

Kwan, H.K., Kwan, B.Y. and Kwan, J.Y. Novel methodologies for spectral classification of exon and intron sequences. *EURASIP Journal on Advances in Signal Processing* 2012;2012(1):1-14.

Liu, G. and Luan, Y. Identification of protein coding regions in the eukaryotic DNA sequences based on Marple algorithm and wavelet packets transform. *Abstract and Applied Analysis* 2014;2014.

Nair, A.S. and Sreenadhan, S.P. A coding measure scheme employing electron-ion interaction pseudopotential (EIIP). *Bioinformation* 2006;1(6):197-202.

Ning, Z., Cox, A.J. and Mullikin, J.C. SSAHA: a fast search method for large DNA databases. *Genome research* 2001;11(10):1725-1729.

Ren, J.*, et al.* Identifying viruses from metagenomic data using deep learning. *Quant Biol* 2020;8(1):64-77.

Sokolova, M. and Lapalme, G. A systematic analysis of performance measures for classification tasks. *Information Processing & Management* 2009;45(4):427-437.

Sun, Y.*, et al.* Prediction of hot spots in protein-DNA binding interfaces based on discrete wavelet transform and wavelet packet transform. *BMC Bioinformatics* 2023;24(1):129.

Tiwari, S.*, et al.* Prediction of probable genes by Fourier analysis of genomic sequences. *Computer applications in the biosciences: CABIOS* 1997;13(3):263-270.

Voss, R.F. Evolution of long-range fractal correlations and 1/f noise in DNA base sequences. *Physical review letters* 1992;68(25):3805.

Yu, F.*, et al.* Deep exploration of random forest model boosts the interpretability of machine learning studies of complicated immune responses and lung burden of nanoparticles. *Sci Adv* 2021;7(22).

Zhang, C.-T., Zhang, R. and Ou, H.-Y. The Z curve database: a graphic representation of genome sequences. *Bioinformatics* 2003;19(5):593-599.

Zhang, Z.-J. DV-Curve: a novel intuitive tool for visualizing and analyzing DNA sequences. *Bioinformatics* 2009;25(9):1112-1117.
